# Mathematical modelling reveals cellular dynamics within tumour spheroids

**DOI:** 10.1371/journal.pcbi.1007961

**Published:** 2020-08-18

**Authors:** Joshua A. Bull, Franziska Mech, Tom Quaiser, Sarah L. Waters, Helen M. Byrne

**Affiliations:** 1 Wolfson Centre for Mathematical Biology, Mathematical Institute, University of Oxford, Oxford, United Kingdom; 2 Roche Pharma Research and Early Development, pRED Informatics, Roche Innovation Centre Munich, Germany; 3 Oxford Centre for Industrial and Applied Mathematics, Mathematical Institute, University of Oxford, Oxford, United Kingdom; University of Southern California, UNITED STATES

## Abstract

Tumour spheroids are widely used as an *in vitro* assay for characterising the dynamics and response to treatment of different cancer cell lines. Their popularity is largely due to the reproducible manner in which spheroids grow: the diffusion of nutrients and oxygen from the surrounding culture medium, and their consumption by tumour cells, causes proliferation to be localised at the spheroid boundary. As the spheroid grows, cells at the spheroid centre may become hypoxic and die, forming a necrotic core. The pressure created by the localisation of tumour cell proliferation and death generates an cellular flow of tumour cells from the spheroid rim towards its core. Experiments by Dorie *et al*. showed that this flow causes inert microspheres to infiltrate into tumour spheroids via advection from the spheroid surface, by adding microbeads to the surface of tumour spheroids and observing the distribution over time. We use an off-lattice hybrid agent-based model to re-assess these experiments and establish the extent to which the spatio-temporal data generated by microspheres can be used to infer kinetic parameters associated with the tumour spheroids that they infiltrate. Variation in these parameters, such as the rate of tumour cell proliferation or sensitivity to hypoxia, can produce spheroids with similar bulk growth dynamics but differing internal compositions (the proportion of the tumour which is proliferating, hypoxic/quiescent and necrotic/nutrient-deficient). We use this model to show that the types of experiment conducted by Dorie *et al*. could be used to infer spheroid composition and parameters associated with tumour cell lines such as their sensitivity to hypoxia or average rate of proliferation, and note that these observations cannot be conducted within previous continuum models of microbead infiltration into tumour spheroids as they rely on resolving the trajectories of individual microbeads.

## Author summary

Tumour spheroids are an experimental assay used to characterise the dynamics and response to treatment of different cancer cell lines. Previous experiments have demonstrated that the localisation of tumour cell proliferation to the spheroid edge (due to the gradient formed by nutrient diffusing from the surrounding medium) causes cells to be pushed from the proliferative rim towards the nutrient-deficient necrotic core. This movement allows inert particles to infiltrate tumour spheroids. We use a hybrid agent-based model to reproduce this data. We show further how data from individual microbead trajectories can be used to infer the composition of simulated tumour spheroids, and to estimate model parameters pertaining to tumour cell proliferation rates and their responses to hypoxia. Since these measurements are possible using modern imaging techniques, this could motivate new experiments in which spheroid composition could be inferred by observing passive infiltration of inert particles.

## Introduction

By the time tumours are clinically detectable *in vivo* they are typically highly heterogeneous in terms of their spatial composition [1]. Tumours contain multiple cell types, including stromal cells (e.g., fibroblasts) and immune cells (e.g., macrophages, T cells) and their growth is sustained by an irregular network of tortuous and immature blood vessels which deliver vital nutrients such as oxygen to the tumour cells. When characterising tumour cell lines or testing new cancer treatments it is important to have a reproducible experimental assay. In such situations, tumour spheroids are widely used due to the predictable manner in which they grow [2].

Tumour spheroids are clusters of tumour cells whose growth *in vitro* is limited by the diffusion of oxygen and other nutrients, such as glucose, from the surrounding medium into the spheroid centre. Other factors which may limit the growth of tumour spheroids include inter-cellular communication, contact sensing, pH levels and/or the circadian clock. In small spheroids, all cells receive sufficient nutrients to proliferate and exponential growth ensues. As a spheroid increases in size, nutrient levels at its centre decrease and may eventually become too low to support cell proliferation, driving cells to halt division and become quiescent. Slower growth of the spheroid will occur until nutrient levels at its centre fall below those needed to maintain cell viability, leading to the formation of a central necrotic core containing dead cells. Growth will continue until the spheroid reaches an equilibrium size at which the proliferation rate of nutrient-rich cells in the outer shell of the spheroid balances the degradation rate of necrotic material at the spheroid centre [2–4]. During necrosis, the cell membrane collapses causing rapid ejection of cell constituents into extracellular space [5], leading to a reduction in cell size as liquid matter disperses into the spheroid.

A wide range of models have been developed to describe the growth and mechanical properties of tumour spheroids [6–8] and organoids [9, 10] and their response to treatment [11, 12]. The simplest models, which include logistic growth and Gompertzian growth, recapitulate the characteristic sigmoid curve describing how the total spheroid volume changes over time [13–15]. These phenomenological models are, however, unable to describe the internal spatial structure of tumour spheroids. More detailed mechanistic models relate the internal spatial structure of the spheroids to the supply of vital nutrients such as oxygen and glucose [16–20], and may be adapted to include the effect of anti-cancer treatments. While some models of spheroid growth account explicitly for factors such as glucose, ATP, pH, and contact inhibition of cell proliferation (e.g., [21]), it is common in mathematical models of tumour spheroids to simplify these complex metabolic processes while retaining the qualitative behaviour of the experimental observations. Most models therefore represent oxygen, glucose and other nutrients via a single diffusible species described variously as “oxygen” or “nutrient”, which is assumed to be vital for the survival and proliferation of tumour cells (e.g., [22–24]).

Agent-based models (ABMs), which resolve individual cells, can also be used to model tumour spheroids. ABMs are often multiscale, linking processes that act at the tissue, cell and subcellular scales. For example, the cell cycle dynamics of individual cells may be modelled via ordinary differential equations (ODEs) at the subcellular scale, may depend on local levels of tissue scale quantities such as oxygen concentration, and may influence cell scale processes such as cell proliferation. ABMs are termed ‘hybrid’ if they combine different modelling approaches. For example, a reaction-diffusion equation describing the spatial distribution of oxygen within a tumour spheroid may be coupled to a stochastic, rule-based cellular automata (CA) model governing the dynamics of individual tumour cells [25]. ABMs can be formulated using on- and off-lattice approaches. On-lattice approaches include rule-based CA models (e.g., [26, 27]) in which each lattice site is typically occupied by at most one cell, and the cellular Potts model [28–30] where individual cells may occupy multiple lattice sites. CA models are generally unable to capture realistic cell shapes or intercellular forces. However, while cellular Potts models permit more realistic cell geometries, they are also constrained by a lattice and do not allow full consideration of mechanical effects.

A weakness of on-lattice models is that cell locations are restricted to discrete lattice sites. By contrast, off-lattice ABMs allow cells to move in a continuous manner through space. Examples of off-lattice ABMs include those which track cell centres (cell-centre approaches) and those which track the cell boundaries (vertex-based approaches). We refer the interested reader to [31] for a comparison of five ABMs (CA, cellular Potts models, overlapping spheres [32], Voronoi tesselation [33] and vertex-based methods).

While many groups develop their own ABMs (e.g., [34]), increasing numbers are using open source software specifically designed for simulating ABMs. The Chaste framework [35, 36] is designed to implement a wide range of ABMs. PhysiCell [37] utilises BioFVM [38] to obtain efficient simulations involving large numbers of diffusing substrates such as oxygen. Morpheus [39] focusses on user-friendliness, with a GUI designed to bypass many of the coding challenges associated with developing agent-based models. Like Chaste, it can implement models using a range of on-lattice or off-lattice frameworks. CompuCell3D [40] provides an intuitive way to implement models using the cellular Potts framework [28]. Other software tools that implement agent-based models include CellSys [41], Biocellion [42], HAL [43] and Timothy [44]. Advantages of these software tools are that code can be more effectively reused and benchmarked, and errors in model implementation are more likely to be identified. Taken together, these frameworks also provide multiple ways of implementing ABMs, so that researchers can choose the framework (or frameworks) best suited to the questions they seek to address.

When developing theoretical models of tumour spheroid growth, a key consideration is the experimental data available to validate and/or parameterise the model. Typically, spheroid experiments generate dynamic data showing how the total tumour volume changes over time. These data may be supplemented by spatially-resolved images of spheroid composition at discrete timepoints [3, 45]. In a series of papers, Dorie *et al*. adopted an alternative approach [46, 47]. They added inert microbeads to the outer edge of well-developed tumour spheroids and collected time-series data showing how the spatial distributions of the microbeads changed over time, as they moved radially inwards, towards the centres of the spheroids. This data is consistent with “passive infiltration”, in which inert particles are advected into the spheroid by tumour cells which are moving down pressure gradients caused by spatial variation in cell proliferation and death. This cellular flow, induced by mechanical stresses within a tumour spheroid, has been proposed as a mechanism by which drugs may exploit advection to enter a tumour more efficiently [48]. Passive infiltration of this type differs from advection by fluid flow, which has previously been implicated as a mechanism by which chemotherapeutic drugs may enter, or be inhibited from entering, tumours [49, 50].

Several authors have developed continuum models to describe Dorie *et al*.’s experiments [51–53]. These continuum models focus on cell populations and, as such, do not resolve individual cells. McElwain and Pettet [51] showed that a possible cause of bead internalisation is pressure gradients caused by differential rates of cell proliferation and death between the proliferative rim and the necrotic core. A modified version of this model [53] also distinguishes between proliferating and quiescent cell populations. Both models assume that dead cells are instantaneously removed from the spheroid and do not occupy space. They also require chemotactic movement of tumour cells in response to the oxygen gradient to reproduce the results from [46]. Thompson and Byrne [52] developed an alternative continuum model to explain the observed infiltration patterns, arguing that differences in infiltration can be explained by non-uniform death and proliferation in the spheroids. Their model assumes for simplicity that the tumour spheroids do not contain a necrotic core. While it reproduces the observed data, their model is unable to reproduce predicted infiltration patterns unless the microbeads are assumed initially to lie strictly inside the tumour spheroid.

In order to reproduce additional data from Dorie *et al*. [46] describing the infiltration of tumour cells labelled with tritiated thymidine (^3^H-labelled cells), McElwain and Pettet [51] assumed that tumour cells move, via chemotaxis, up spatial gradients in the oxygen concentration, and neglected proliferation of the labelled cells. Thompson and Byrne [52] assumed that labelled cells proliferate, but as with their microbead model the labelled cells had to be initially placed within the tumour spheroid to reproduce infiltration results. By varying chemotaxis coefficients between the subpopulations, the authors reproduced the observed infiltration patterns. As they use a continuum framework, these models cannot resolve individual microbeads or labelled cells.

Inspired by the original experiments of Dorie *et al*. [46], and the increasing use of ABMs, we develop an off-lattice hybrid ABM to describe the growth of tumour spheroids and their infiltration by microbeads and ^3^H-labelled tumour cells. This enables us to resolve the trajectories of individual cells and microbeads within the model, something which continuum models are unable to do. We show further how observations of microbead trajectories can be used to identify the composition of a simulated spheroid and estimate parameters associated with cell cycle duration and cell responses to hypoxia. After verifying that the model reproduces the spatio-temporal dynamics of tumour spheroids growing in free suspension *in vitro*, we use it to simulate their infiltration by inert microbeads and labelled tumour cells (see Supporting Information, S5 Appendix: Replication of ^3^H-labelled cells experiments), obtaining good agreement with Dorie *et al*.’s experimental results. A parameter sensitivity analysis reveals how the growth rate, size and spatial structure of the spheroids change as we vary key model parameters. We show how spheroids with the same equilibrium size may differ in their spatial organisation. We conclude by showing how dynamic data describing the trajectories of individual microbeads, which cannot be resolved using continuum models, can be used to infer the composition of simulated tumour spheroids, and also to estimate model parameters pertaining to tumour cell proliferation rates and cell sensitivity to hypoxia.

## Materials and methods

### Model overview

We develop a hybrid agent-based mathematical model to describe the *in vitro* growth of tumour spheroids in response to an externally-supplied nutrient, here taken to be oxygen. We use the model to simulate spheroid infiltration by inert microbeads and ^3^H-labelled cells. Our model is implemented within Chaste (Cancer, Heart and Soft Tissue Environment, available at https://www.cs.ox.ac.uk/chaste/), an open source simulation package designed to solve computationally demanding, multiscale problems that arise in biology and medicine [35, 36]. We choose this framework because it provides an efficient means of implementing off-lattice ABMs, and has previously been used to simulate multicellular spheroids [35, 54]. The extensions to the framework described in this paper will be made available in a subsequent release of the Chaste software.

**Fig 1:**
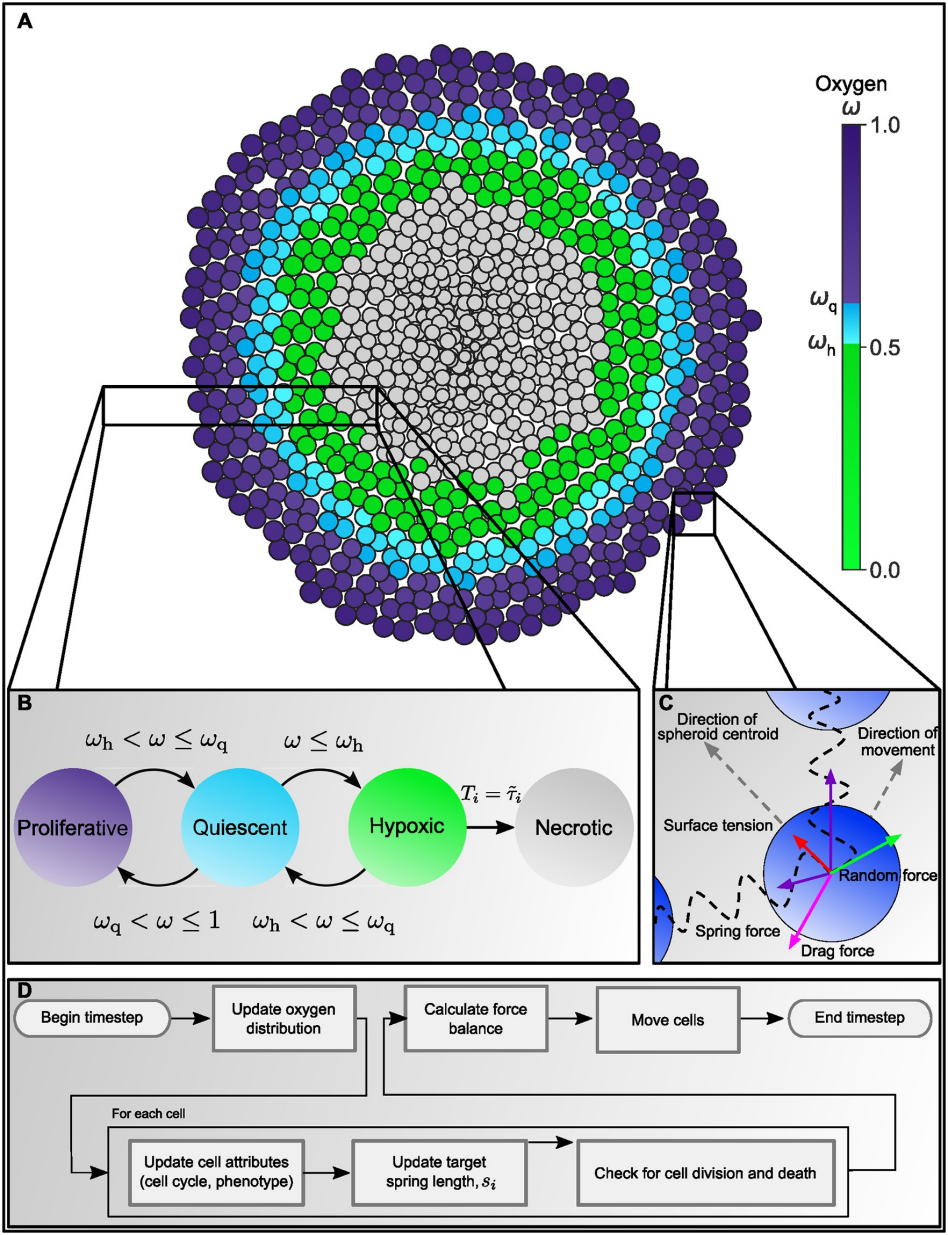
Overview of our agent-based model for the growth of tumour spheroids. A: As oxygen *ω* diffuses from the outer spheroid boundary, it is consumed by live cells. Consequently, the oxygen concentration at the centre decreases as the spheroid increases in size. We use the oxygen distribution to distinguish up to four different regions, or compartments, within a spheroid: a well-oxygenated rim, where *ω*_*q*_ ≤ *ω* ≤ 1, contains proliferating cells; a quiescent compartment, where *ω*_*h*_ ≤ *ω* < *ω*_*q*_, contains non-proliferating viable cells; a hypoxic compartment, where *ω* ≤ *ω*_*h*_, contains non-proliferating viable cells which will become necrotic if they remain hypoxic for longer than a prescribed time period; and a necrotic compartment, containing dead cells which degrade over time. B: Schematic showing how the way in which cells switch between different compartments depends on the local oxygen concentration. C: Schematic showing the forces which act on individual cells to determine cell movement. All nodes experience spring forces due to interactions with their neighbours, a random force which represents local fluctuations in the cell environment, and a drag force which resists cell movement. Boundary nodes also experience a surface tension force which is directed inwards, towards the spheroid centroid, and which resists spheroid expansion. D: Flowchart summarising how the ABM is updated on each timestep—see main text and Supporting Information, S2 Appendix: Algorithm for updating the cell cycle, for details.

A schematic highlighting the key features of our hybrid ABM is presented in Fig 1. Individual cells are represented using an off-lattice, cell-centre model, and cell movement is determined via consideration of the force balances on each cell and assuming that inertial effects can be neglected (Panel C of Fig 1 indicates those forces which act on cells). Interactions between cells are modelled by connecting cell centres with springs (Fig 1, Panel C), which simulate both intercellular adhesion and volume exclusion. Through appropriate choice of the spring lengths associated with each cell, the model includes a notion of cell size which can be adjusted to account for size differences between developed cells and those which have just proliferated, or which are decaying due to necrosis.

We distinguish two types of agent: *tumour cells* and *microbeads*. Tumour cell behaviours (e.g., cell cycle progression, quiescence and cell death) depend on the local oxygen concentration, which is determined by a reaction-diffusion equation accounting for oxygen consumption by viable tumour cells and oxygen diffusion from the spheroid boundary towards its centre. The flowchart at the bottom of Fig 1 shows how simulation results are generated. We note that while this model can simulate spheroid growth in three dimensions, here we restrict attention to 2D simulations to reduce computational time.

In the rest of this Section, we introduce the agent-based model we have developed to simulate tumour spheroid growth and microbead infiltration. We first describe the PDE used to calculate the oxygen distribution throughout the spheroid, and then discuss the impact oxygen concentration has on tumour cell behaviour. We outline the rules used to implement proliferation and death, and the forces which act on different types of agent to determine their movement. Finally, we summarise the different simulations conducted using this model and the rules used to initialise them.

### Oxygen distribution

While we model cells and microbeads as discrete entities, we assume that the concentration of oxygen *ω*(**x**, *t*) is continuous and can be described via a reaction-diffusion equation, with oxygen consumption by live tumour cells modelled by placing point sinks at the centres of viable cells. Written in dimensional form, the equation governing the spatio-temporal evolution of the oxygen concentration is thus

$$\begin{array}{c} \frac{\partial \omega }{\partial t}=D_{\omega }\nabla^{2}\omega -\kappa \omega \sum_{i}\delta \left(\text{x}-{\text{x}}_{i}\right) \end{array}$$ (1)

for **x** ∈ Ω, where **x**_*i*_ is the location of viable cell *i*, the parameter *D*_*ω*_ is the assumed constant diffusion coefficient of oxygen, *κ* is the oxygen consumption rate and Ω is the simulation domain, which we take to be a square domain large enough to fully enclose the spheroid. *δ*(**x**) is the delta function (*δ*(**x**) = 1 when **x** = 0; *δ*(**x**) = 0 otherwise).

Eq (1) is solved subject to Dirichlet boundary conditions, which are prescribed on the domain boundary, and suitable initial conditions. We assume that oxygen is maintained at a constant level in the culture medium surrounding the tumour spheroid and, hence, by continuity that the oxygen concentration on the spheroid boundary is also maintained at this constant value, *ω*_∞_.

Since the timescale for oxygen diffusion (seconds) is much shorter than the timescale for cell proliferation (hours), when we nondimensionalise Eq (1), we make the standard, quasi-steady state approximation (see S3 Appendix: Non-dimensionalisation of oxygen equation for details) and solve instead

$$\begin{array}{c} 0=D_{\omega }\nabla^{2}\omega -\kappa \omega \sum_{i}\delta \left(\text{x}-{\text{x}}_{i}\right). \end{array}$$ (2)

We solve Eq (2) on a regular tetrahedral finite element mesh which spans Ω, a square domain large enough to contain all the cell centres. We fix *ω* = *ω*_∞_ at any nodes of the mesh which lie outside the spheroid.

### Tumour cell phenotypes

While microbeads do not consume oxygen and are unaffected by the oxygen concentration, the phenotype of tumour cells depends on their local oxygen concentration. We distinguish four types of tumour cell behaviour by introducing the following phenotypes (see Fig 1):

- A tumour cell is **proliferative** if its local oxygen concentration exceeds a threshold value, *ω*_q_.
- If *ω*(**x**, *t*) ≤ *ω*_q_, then the cell becomes **quiescent** and immediately pauses its cell cycle. If *ω* increases above *ω*_*q*_ then the cell immediately becomes proliferative and resumes its cell cycle.
- If the oxygen concentration falls below a second, hypoxic threshold, 0 ≤ *ω*_h_ ≤ *ω*_q_, then the cell immediately becomes **hypoxic**. A hypoxic cell can re-enter the quiescent compartment if its oxygen concentration rises back above *ω*_h_. However, if a cell remains hypoxic for sufficiently long (${\tilde{\tau }}_{i}$ hours) then it will irreversibly become **necrotic** [55].
- A **necrotic** cell is dead, and no longer consumes oxygen. Necrotic cells continue to occupy space for approximately $\bar{\tau }$ hours before being removed from the simulation.

We also simulate cells labelled with tritiated thymidine (^3^H-labelled cells) to replicate experimental results from Dorie *et al*. [46] (see Supporting Information, S5 Appendix: Replication of ^3^H-labelled cells experiments). In this model ^3^H-labelled cells are assumed to behave in the same way as other tumour cells. They adopt the same phenotypes as unlabelled cells in response to local environmental cues. The only difference between ^3^H-labelled cells and other tumour cells is the presence of a label which which is passed on to their daughter cells, enabling their lineage to be tracked.

### Tumour cell proliferation and death

For each individual cell *i*, proliferation and death are determined by two subcellular dependent variables: the *cell cycle time* denoted by *T*_*i*_, which tracks a cell’s progress through the cell cycle, and the *hypoxia time* denoted by $\tilde{T_{i}}$ which determines whether a cell is sufficiently hypoxic to become necrotic. These internal timers move at a rate which depends on the local oxygen concentration *ω*(**x**, *t*). Pseudocode describing how the cell cycle is updated is presented in the Supporting Information, S2 Appendix: Algorithm for updating the cell cycle.

#### Cell proliferation

At birth, the cell cycle time of cell *i* is initialised such that *T*_*i*_ = 0, and the cell is assigned a cell cycle duration *τ*_*i*_ drawn from a uniform distribution *U*(0.75*τ*, 1.25*τ*) where the parameter *τ* defines the average cell cycle length. The distribution was chosen to be sufficiently wide to ensure that cell cycles do not become artificially synchronised over time, while ensuring that *τ* remains a good descriptor of the average cell cycle duration. If cell *i* is at position **x** at time *t* then its cell cycle evolves as follows:

$$\begin{array}{c} \frac{dT_{i}}{dt}=H\left(\omega \left(\text{x},t\right)-\omega_{q}\right) \end{array}$$ (3)

where *ω*(**x**, *t*) is the local oxygen concentration at time *t* and location **x**, H is the Heaviside step function ($H(\omega -\omega_{q})=1$ if *ω* > *ω*_q_; $H(\omega -\omega_{q})=0$ otherwise). When *ω* ≤ *ω*_q_, the cell cycle pauses and the cell remains dormant until either *ω* increases above the threshold (and progress through the cell cycle resumes) or the cell becomes necrotic (details of this process are described in the next section). When *T*_*i*_ = *τ*_*i*_, cell division occurs and a daughter cell is placed half a cell diameter away from the parent cell centre in a randomly chosen direction. Both cells are assigned new cell cycle durations. Their cell cycle times are reset to 0 and evolve according to Eq (3). The resting spring length of the new cells is adjusted to account for the reduced size of the new cells (for details, see the description of the spring force laws below).

#### Cell death

Hypoxic cells at locations where *ω* ≤ *ω*_h_ undergo necrotic cell death if they remain hypoxic for longer than a threshold time ${\tilde{\tau }}_{i}$. In a manner similar to that used to model cell cycle progress, ${\tilde{\tau }}_{i}$ is drawn from a uniform distribution $U(0.75\;\tilde{\tau },1.25\;\tilde{\tau })$ where $\tilde{\tau }$ is the average duration for which a cell is hypoxic before it becomes necrotic. As for the parameter *τ*, the distribution around $\tilde{\tau }$ enables us to account for stochastic fluctuations in cell properties and also to suppress the emergence of artificial oscillations in the number of necrotic cells caused by multiple cells simultaneously becoming necrotic.

Each cell is assigned an internal hypoxia time, $\tilde{T_{i}}$, which progresses when a cell is hypoxic and evolves as follows:

$$\begin{array}{c} \frac{d\tilde{T_{i}}}{dt}=H\left(\omega_{h}-\omega \left(\text{x},t\right)\right), \end{array}$$ (4)

with $\tilde{T_{i}}=0$ at the onset of hypoxia. If the cell moves or the oxygen distribution changes so that *ω*(**x**_*i*_, *t*) > *ω*_h_ then we set $\tilde{T_{i}}=0$, indicating that the cell has received sufficient nutrient to prevent cell death. A cell becomes necrotic when $\tilde{T_{i}}={\tilde{\tau }}_{i}$. It is then irreversibly marked for cell death. Once a tumour cell has become necrotic, it is no longer viable and no longer progresses through the cell cycle. It continues to occupy space, but reduces in size until it is removed from the simulation over a period of ${\bar{\tau }}_{i}$ hours where ${\bar{\tau }}_{i}$ is drawn from a uniform distribution $U(0.75\;\bar{\tau },1.25\;\bar{\tau })$ and $\bar{\tau }$ is the average duration of necrosis. As with the cell cycle duration and the time threshold which triggers necrosis, the range of this distribution is estimated and reflects variation in the process of cell degradation. Details on how size reduction is implemented are included below.

### Force balance

We use Newton’s second law to derive the equations of motion for cells and microbeads. In the over-damped limit, we neglect inertial effects and obtain the following force balances for cell *i* and microbead *j* respectively:

$$\begin{array}{c} \nu \frac{d{\text{x}}_{\text{i}}}{dt}={\text{F}}_{i}^{m}+{\text{F}}_{i}^{r}+{\text{F}}_{i}^{s}, \end{array}$$ (5)

$$\begin{array}{c} \nu \frac{d{\text{x}}_{j}}{dt}={\text{F}}_{j}^{m}+{\text{F}}_{j}^{r}. \end{array}$$ (6)

In Eqs (5) and (6) we assume that the drag forces on cell *i* and microbead *j* are proportional to their velocities, the constant *ν* denoting the drag coefficient. We denote the mechanical force by ${\text{F}}_{i}^{m}$, random forces by ${\text{F}}_{i}^{r}$, and surface tension forces by ${\text{F}}_{i}^{s}$. Mechanical and random forces act on cells and microbeads, whereas surface tension forces only act on cells. Functional forms for these forces are introduced below.

The timestep *dt* used to generate numerical solutions is taken to be 1/120 of a dimensionless time unit, which is equivalent to 30 seconds (see Supporting Information, S7 Appendix: Table of parameters).

#### Mechanical forces, ${\text{F}}_{i}^{m}$ (cells and microbeads.)

The mechanical spring force acting on a cell or microbead is the net force exerted on it by its neighbours. We assume that cells/microbeads *i* and *j* only interact if the distance between their centres is less than a fixed value, *R*_int_. In more detail, and following the overlapping sphere approach outlined in [32, 33, 56, 57], if |**x**_**i**_ − **x**_**j**_| < *R*_int_ then the interaction force between cells/microbeads *i* and *j* is parallel to the vector **x**_**i**_ − **x**_**j**_ connecting their centres. The magnitude of the force depends on the sizes of the cells/microbeads and the distance between them. While agents in this model are represented as points, each point has an associated size which is implemented by adjusting the resting spring lengths for each cell/microbead. The resting spring length between two nodes, *s*_*i*,*j*_, is the sum of the equilibrium springs for each cell (*s*_*i*,*j*_ = *s*_*i*_ + *s*_*j*_). For most cells *i*, *s*_*i*_ = *R*_Cell_ is a constant which is approximately equal to the radius of a cell. For newborn and necrotic cells, cell growth or shrinkage may mean that *s*_*i*_ < *R*_Cell_. These processes are described below.

If the distance between the cell centres of cells *i* and *j* is greater than *s*_*i*,*j*_ then the cells experience an attractive force representing intercellular adhesion, but if the distance is less than *s*_*i*,*j*_ then the force is instead repulsive and models volume exclusion. The net force acting on a cell or microbead *i* at location **x**_*i*_ due to mechanical forces is the sum of the contributions over all cells and microbeads *j* within radius *R*_int_:

$$\begin{array}{c} {\text{F}}_{i}^{m}=\sum_{\left\{j\;\;|\;\;|{\text{x}}_{i}-{\text{x}}_{j}|\leq R_{\text{int}}\right\}}{\text{F}}_{i,j}^{m}. \end{array}$$ (7)

where ${\text{F}}_{i,j}^{m}$ is the mechanical force between cells *i* and *j*. This force always points in the direction of the vector between the cells. The magnitude of ${\text{F}}_{i,j}^{m}$ is defined as follows:

$$\begin{array}{c} |{\text{F}}_{i,j}^{m}|=\{\begin{array}{c} \mu s_{i,j}\;\text{log}\;\left(1+\frac{x}{s_{i,j}}\right)\;\text{if}\;x<0\left(\text{Repulsive}\right)\\ \mu xs_{i,j}\;\text{exp}\;\left(-\lambda \frac{x}{s_{i,j}}\right)\;\text{if}\;x\geq 0\left(\text{Adhesive}\right) \end{array} \end{array}$$ (8)

where *x* = |**x**_*i*_ − **x**_*j*_| − *s*_*i*,*j*_ is the overlap between cells *i* and *j*, the parameter *μ* represents the spring stiffness and the parameter λ determines the strength of intercellular adhesion between neighbouring cells. A sketch showing how $|{\text{F}}_{i,j}^{m}|$ changes as *x* varies is shown in the Supporting Information, S1 Appendix: Spring force magnitude. The adhesive force defined in Eq (8) grows stronger as the cell centres draw closer, since more intercellular bonds form as the surface area of contact between the cells increases [32, 58].

Inert microbeads also interact with neighbouring cells and microbeads. Since microbeads do not adhere to each other we modify the mechanical force described in Eq (8) when *i* and *j* are both microbeads as follows:

$$\begin{array}{c} |{\text{F}}_{i,j}^{m}|=\{\begin{array}{cc} \mu_{\text{bead}}s_{i,j}\;\text{log}\;\left(1+\frac{x}{s_{i,j}}\right) & \text{if}\;x<0\\ 0 & \text{if}\;x\geq 0 \end{array}. \end{array}$$ (9)

where *μ*_bead_ is the spring stiffness associated with microbeads. Beads therefore resist being compressed, but do not adhere to other beads.

With the exception of newly divided cells and necrotic cells, we assume that *s*_*i*_ = *R*_Cell_ for cells and microbeads (as the radius of a microbead is comparable to that of a cell). For convenience, we rescale lengths according to this lengthscale, assuming that 1 cell diameter = 2*R*_Cell_ = 20*μ*m. When a cell divides, the radius of the new cells is set to $s_{i}=\frac{R_{\text{Cell}}}{2}$ and increases linearly over the course of one hour until *s*_*i*_ = *R*_Cell_. For necrotic cells, *s*_*i*_ decreases linearly over the course of ${\bar{\tau }}_{i}$ hours until it reaches 0 and the cell is removed from the simulation. The associated spring constant of springs attached to a necrotic cell is reduced linearly at the same rate, representing a weakening intercellular force between a necrotic cell and neighbouring cells as the necrotic cell degrades. These reductions in cell size are incorporated in the pseudocode in the Supporting Information, S2 Appendix: Algorithm for updating the cell cycle.

#### Random forces, ${\text{F}}_{i}^{r}$ (cells and microbeads)

We assume that cells and microbeads experience random forces due to heterogeneity in the surrounding environment and that the random force, ${\text{F}}_{i}^{r}=\left(F_{x}^{r},F_{y}^{r}\right)$, acting on cell/microbead *i* during the timestep *dt* is given by:

$$\begin{array}{c} {\text{F}}_{i}^{r}=\sqrt{2Ddt}\;\xi . \end{array}$$ (10)

In Eq (10), *D* is a diffusion coefficient and ***ξ*** = (*ξ*_*x*_, *ξ*_*y*_) where *ξ*_*x*_ and *ξ*_*y*_ are random variables drawn from a standard normal distribution.

#### Surface tension forces, ${\text{F}}_{i}^{s}$ (boundary cells only)

Cells on the spheroid boundary experience a force, ${\text{F}}_{i}^{s}$, of the form

$$\begin{array}{c} {\text{F}}_{i}^{s}=-\beta {\hat{\text{x}}}_{i}, \end{array}$$ (11)

where ${\hat{\text{x}}}_{i}$ is a unit vector pointing in the direction of the line connecting cell *i* on the boundary with the spheroid centroid, and the parameter *β* determines the strength of the surface tension force. We define boundary cells as those which belong to the *α*-shape of the set of cell centres, where *α* = *R*_Cell_ [59]. *α*-shapes generalise the concept of a convex hull to permit concave boundaries, and can be understood as the set of points which make contact with a ball of radius *α* rolled around the edge of the spheroid.

### Simulations

We use our ABM to perform three types of simulations: (i) growth of a tumour spheroid, (ii) microbead infiltration into a well-developed tumour spheroid, and (iii) infiltration of ^3^H-labelled cells into a well-developed tumour spheroid (see Supporting Information, S5 Appendix: Replication of ^3^H-labelled cells experiments).

Simulations of spheroid growth are initialised by uniformly distributing 300 cells in a circle of radius 5 cell diameters. The cells are then are allowed to grow for 300 hours, which is sufficient for them to attain a steady state for most parameter regimes examined here. At the start of each simulation, all cells are randomly assigned a cell cycle time *T*_*i*_ from a uniform distribution *U*(0, 0.75*τ*). This ensures that the cell cycles are not synchronised. When performing parameter sensitivity analyses, three model parameters were varied: the average cell cycle length, *τ*, the oxygen threshold for quiescence, *ω*_q_, and the oxygen threshold for hypoxia, *ω*_h_. We focus on these parameters as they have a significant effect on tumour spheroid composition, and are known to vary between different tumour cell lines.

For microbead infiltration simulations, 100 microbeads were distributed randomly around the spheroid edge after 300 hours, and the simulation allowed to evolve for a further 100 hours, reproducing the conditions under which Dorie *et al*. [46] tracked microbeads experimentally. For ^3^H-labelled cell experiments, 50 cells on the spheroid boundary were randomly selected after 300 hours and marked with a label which was transmitted on cell division but did not affect cell behaviour.

For details regarding the parameter values used here, we refer the reader to the Supporting Information, S7 Appendix: Table of parameters. Throughout this paper, when estimates of simulated tumour cell phenotype compartment widths are stated for a particular simulation realisation, this width is calculated as the difference in radial distance from the spheroid centroid between the innermost and outermost cells with that phenotype.

### Units and non-dimensionalisation

We refer to non-dimensionalised times, distances and concentrations. Lengths are non-dimensionalised with the diameter of a cell, so a dimensionless distance of 1.0 corresponds to approximately 20 *μ*m. Times are rescaled so one dimensionless time unit corresponds to 1 hour. Concentrations are rescaled with the externally-supplied concentration of oxygen, so a dimensionless concentration of 1.0 equates to approximately 3.298 × 10^−6^ m^3^ kg^−1^ in dimensional units (using Henry’s law to convert from partial pressure to concentration and following [12, 60]).

## Results

We use our hybrid agent-based model to simulate tumour spheroids whose growth dynamics and spatial structure replicate those of spheroids cultured *in vitro*. We first demonstrate that this model reproduces the sigmoidal growth curves observed in *in vitro* experiments. we then show that when microbeads are added to the *in silico* spheroids their infiltration patterns are qualitatively similar to those described by Dorie *et al*. [46]. By performing a systematic parameter sensitivity analysis, we show how varying the oxygen thresholds *ω*_q_ and *ω*_h_ and the average cell cycle length *τ* can generate *in silico* tumour spheroids with similar growth dynamics and different spatial compositions. Finally, we demonstrate that resolving the trajectories of individual microbeads within our agent-based model provides sufficient information to predict spheroid composition and infer certain model parameter values.

### Model Validation

**Fig 2:**
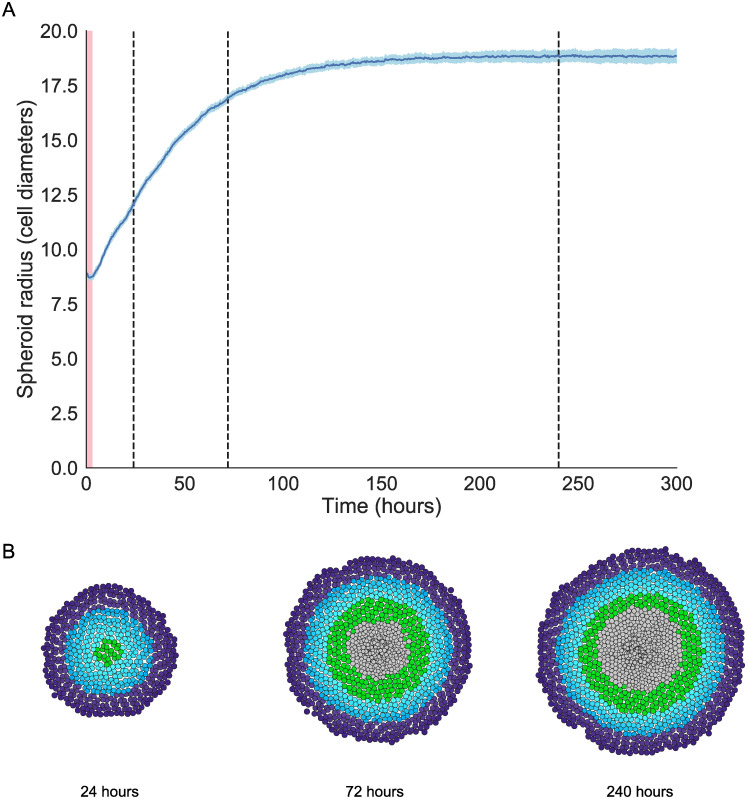
Model validation I: reproducing spheroid growth dynamics. A: Saturating growth curve for one representative parameter set (Parameters: *ω*_q_ = 0.5, *ω*_h_ = 0.3, *τ* = 16, $\tilde{\tau }=8$) showing total spheroid radius over time (mean and standard deviation of 40 realisations). The spheroid radius decreases during an initial burn in period as cells relax from their initial positions to form a densely packed cluster of cells, highlighted in red (*t* = 0 to *t* = 4). After this burn in period, the simulations show initial rapid growth followed by growth saturation when spheroid size is sufficiently large. B: Simulation snapshots showing the spheroid composition after 24, 72 and 240 hours for the parameter set in A. Times of snapshots are marked with a dashed line in A. During the rapid growth phase the spheroid consists primarily of proliferative and quiescent cells, and the reduction in spheroid growth rate is associated with the formation of the necrotic core.

We first show that our ABM qualitatively reproduces the dynamics and changing spatial structure that characterises spheroid growth *in vitro*. Four parameters relating to the proliferation and death rates of tumour cells were varied: the oxygen thresholds at which cells become quiescent or hypoxic, *ω*_q_ and *ω*_h_, the average cell cycle duration *τ* and the average duration of hypoxia before cell death, $\tilde{\tau }$ (ranges given in Supporting Information, S7 Appendix: Table of parameters). Typical simulation results generated using representative parameter values (*ω*_q_ = 0.5, *ω*_h_ = 0.3, *τ* = 16, $\tilde{\tau }=8$) are presented in Fig 2. We observe a short burn-in period of approximately 2-4 hours, during which the cells move from their initial positions to form a densely packed spheroid. Thereafter the tumour radius grows rapidly, slowing when nutrient levels at its centre become too low to support cell proliferation, and saturating when the rate at which nutrient-rich cells proliferate balances the rate at which necrotic cells are degraded [3]. For the parameter values chosen here, the tumour reached its steady state approximately 150 hours after beginning the simulation, with a steady state radius comparable to those observed in the experiments by Dorie *et al*. [46].

**Fig 3:**
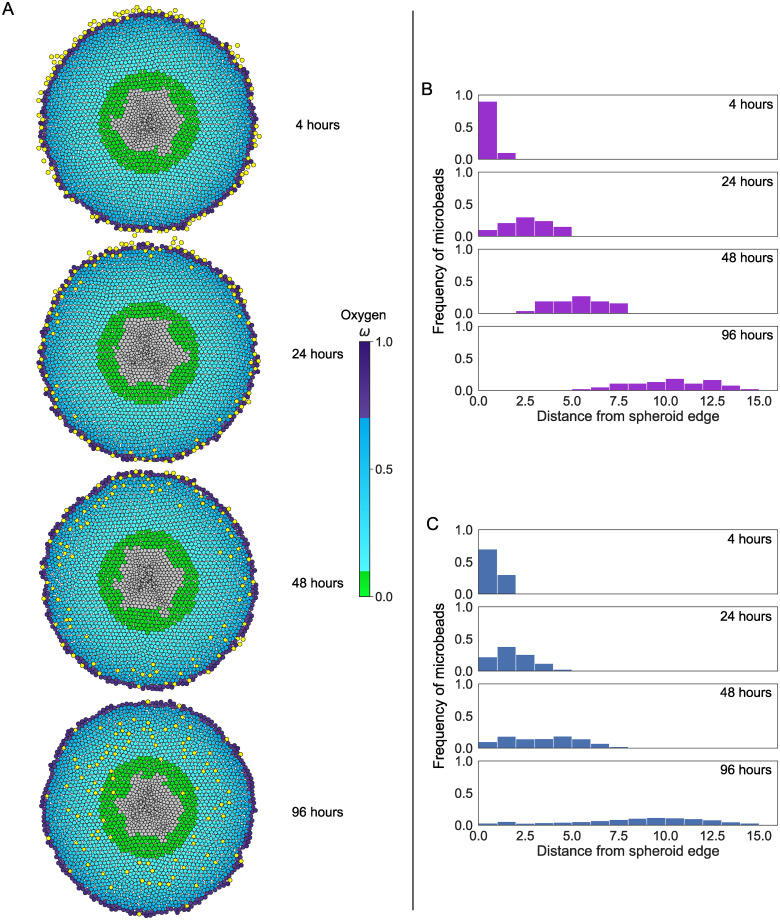
Model validation II: reproducing microbead infiltration patterns. A: Realisations of a typical simulation showing microbead infiltration (yellow circles) over time. Microbeads were added to the spheroid after 300 simulation hours, and permitted to infiltrate the spheroid for a further 100 hours. Times stated are times after microbeads were added. Tumour cells are coloured according to their phenotype (see Fig 1). Parameter values *ω*_q_ = 0.7, *ω*_h_ = 0.1, *τ* = 8, $\tilde{\tau }=16$. B: Frequency histograms showing the experimentally observed distribution of microbeads in spheroids (reproduced from Fig 7 of Dorie *et al*. [46]. C: Frequency histograms showing the distribution of microbeads in spheroids generated from this model, averaged over 40 iterations of the parameter set in A. For this parameter set, the distribution of microbeads closely resembles that in Panel B. Histograms showing the distributions obtained for different parameter sets can be seen in S4 Appendix: Microbead infiltration histograms for other parameter combinations. Results comparing the model with the ^3^H-labelled data from [46] can also be found in the Supporting Information, S5 Appendix: Replication of ^3^H-labelled cells experiments.

Fig 3 demonstrates that the model reproduces the qualitative behaviour of the radial distribution of infiltrating microbeads which was observed in [46]. These distributions resemble a wave which becomes increasingly dispersed as it moves radially inwards from the spheroid edge. The parameter values used in Fig 3 (*ω*_q_ = 0.7, *ω*_h_ = 0.1, *τ* = 8, $\tilde{\tau }=16$) were chosen to produce a distribution which matches that described in [46]. We remark that spheroids generated using different parameter values may produce microbead distributions which are not visually distinguishable (see Supporting Information, S4 Appendix: Microbead infiltration histograms for other parameter combinations). In addition to reproducing the dynamics of infiltrating microbeads in the experimental data described in [46], this model can also reproduce the observed distributions of ^3^H-labelled cells (see Supporting Information, S5 Appendix: Replication of ^3^H-labelled cells experiments). In particular, we note that, as for microbead infiltration patterns, ^3^H-labelled cell infiltration patterns are strongly affected by variation of the parameters which control tumour cell proliferation and death. We can identify regimes in which the distribution of ^3^H-labelled tumour cells closely resembles that described by Dorie *et al*. In contrast to previous models of ^3^H-labelled cell infiltration, no additional mechanisms are required to describe differences in the distributions of ^3^H-labelled cells and microbeads. Instead, differences in their distributions can be attributed to proliferation of the ^3^H-labelled cells which causes the peak of the distribution to remain localised near the spheroid boundary where oxygen levels and, hence, cell proliferation rates are maximal. (For further details, see Supporting Information, S5 Appendix: Replication of ^3^H-labelled cells experiments.)

### Spheroids with the same equilibrium size may have different spatial compositions

**Fig 4:**
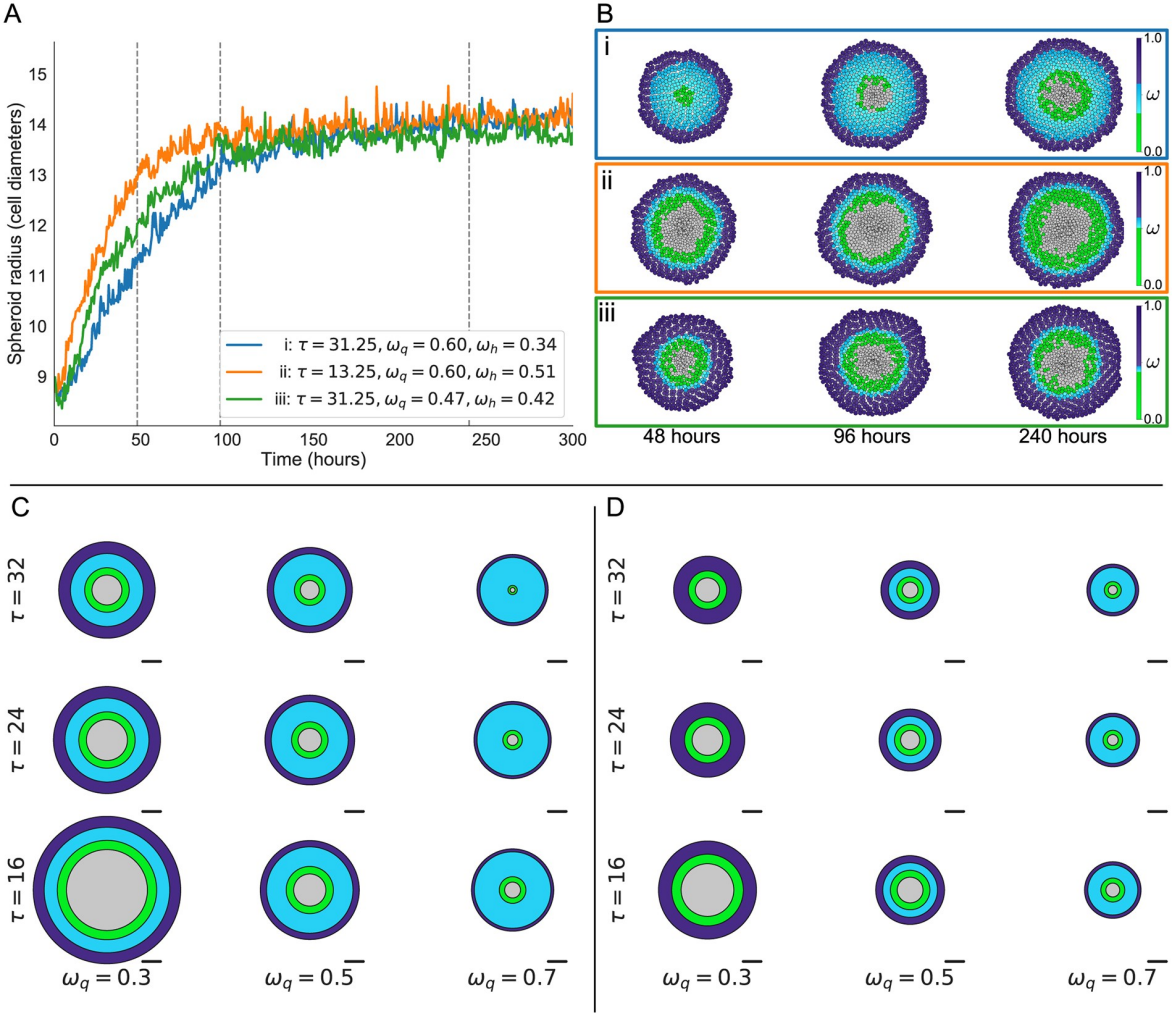
Spheroids with similar growth dynamics can have different compositions. A: Tumour radius over time for three spheroids generated from different parameter sets which give rise to spheroids with approximately the same steady state radius. B: Snapshots of the simulations in A. Cells are coloured according to their oxygen concentration and phenotype. By varying the thresholds *ω*_q_ and *ω*_h_, and the growth rate *τ*, we can generate spheroids with similar growth dynamics and steady state radii which have different internal compositions. Spheroids i and ii have the same *ω*_q_, but differ in *ω*_h_ and *τ*. Spheroids i and iii have the same *τ*, but differ in *ω*_q_ and *ω*_h_. Spheroid i (blue line) contains a large number of quiescent cells and has a slow growth rate, but has a low oxygen threshold before cells become hypoxic. Cells in Spheroid ii (yellow line) proliferate more quickly than in Spheroid i, but since *ω*_h_ is higher the spheroid has a larger necrotic core at steady state. Spheroid iii (green line) also has only a narrow quiescent region, but possesses a thicker proliferative rim than spheroid ii. C and D: Average spheroid sizes and compositions change as *ω*_q_ and *τ* vary, averaged over 50 repetitions for each parameter set (with $\tilde{\tau }=8$ and *ω*_h_ = 0.1 (C) or *ω*_h_ = 0.3 (D)). Scale bars are 10 cell diameters in length.

We systematically vary three key model parameters: the average cell cycle length, *τ*, and the oxygen thresholds for quiescence and hypoxia, *ω*_q_ and *ω*_h_. We focus on these parameters as they are directly linked with the spheroid growth rate, the proportion of cells which are part of the proliferative rim, and the rate at which non-proliferating cells undergo cell death. As these parameters vary, we can generate spheroids which exhibit a range of behaviours (see Fig 4).

Panel A of Fig 4 shows spheroid growth curves for simulations generated from three different parameter sets. Each simulated spheroid reaches a steady state radius of approximately 14 cell diameters. Simulations i and ii are both generated with *ω*_q_ = 0.6, and different values of *τ* and *ω*_h_. While the cells in Spheroid ii proliferate more often than those in Spheroid i, this effect is offset by the change in *ω*_h_ which causes cells in Spheroid ii to become necrotic at higher oxygen levels than those in Spheroid i. This difference can be seen in Fig 4, Panel B, which shows that Spheroid ii has a more pronounced necrotic core and thinner quiescent region than Spheroid i.

Similarly, Spheroid i and Spheroid iii have the same average cell cycle length *τ* = 31.25, but differ in both *ω*_q_ and *ω*_h_. While *ω*_q_ has been lowered from *ω*_q_ = 0.6 to *ω*_q_ = 0.47, they realise similar equilibrium sizes because *ω*_h_ increases from *ω*_h_ = 0.34 to *ω*_h_ = 0.42. These parameter changes cause Spheroid iii to have a thicker proliferating rim and narrower quiescent region than Spheroid i.

By appropriately tuning the proliferation rate and oxygen thresholds at which cells become quiescent or die, we can generate synthetic spheroids of the same equilibrium size which possess different internal compositions. This suggests that spheroid composition cannot be predicted by observing the overall growth dynamics alone. Panels C and D of Fig 4 show the effect of varying *τ* and *ω*_q_ on the steady state size and composition of tumour spheroids, averaged over 50 stochastic repetitions for each parameter combination with *ω*_h_ = 0.1 (Panel C) or *ω*_h_ = 0.3 (Panel D). For each fixed value of *ω*_h_, lowering *ω*_q_ causes the proliferating rim to become wider and the spheroid to become larger. Decreasing the average cell cycle length *τ* causes the spheroid radius to increase, but this is due mainly to increases in the hypoxic and necrotic volumes of the spheroid.

This result indicates that data on tumour size alone are not predictive of tumour composition. Additional data describing spatial structure and heterogeneity in tumour composition could improve the predictive power of models, particularly their ability to simulate responses to treatments such as radiotherapy which affect hypoxic and well-oxygenated cells differently.

### Bead trajectories change as spheroid composition and growth dynamics vary

One advantage of using an agent-based framework rather than a continuum model to simulate passive infiltration [51–53] is that the trajectories of individual microbeads can be tracked in addition to the bead distribution (given by the infiltration histograms in Fig 3). We can use these trajectories to better understand why microbead distributions disperse while travelling radially inwards, but also to move beyond the original experiments in [46] to use beads to predict both spheroid composition and parameters associated with individual spheroids.

**Fig 5:**
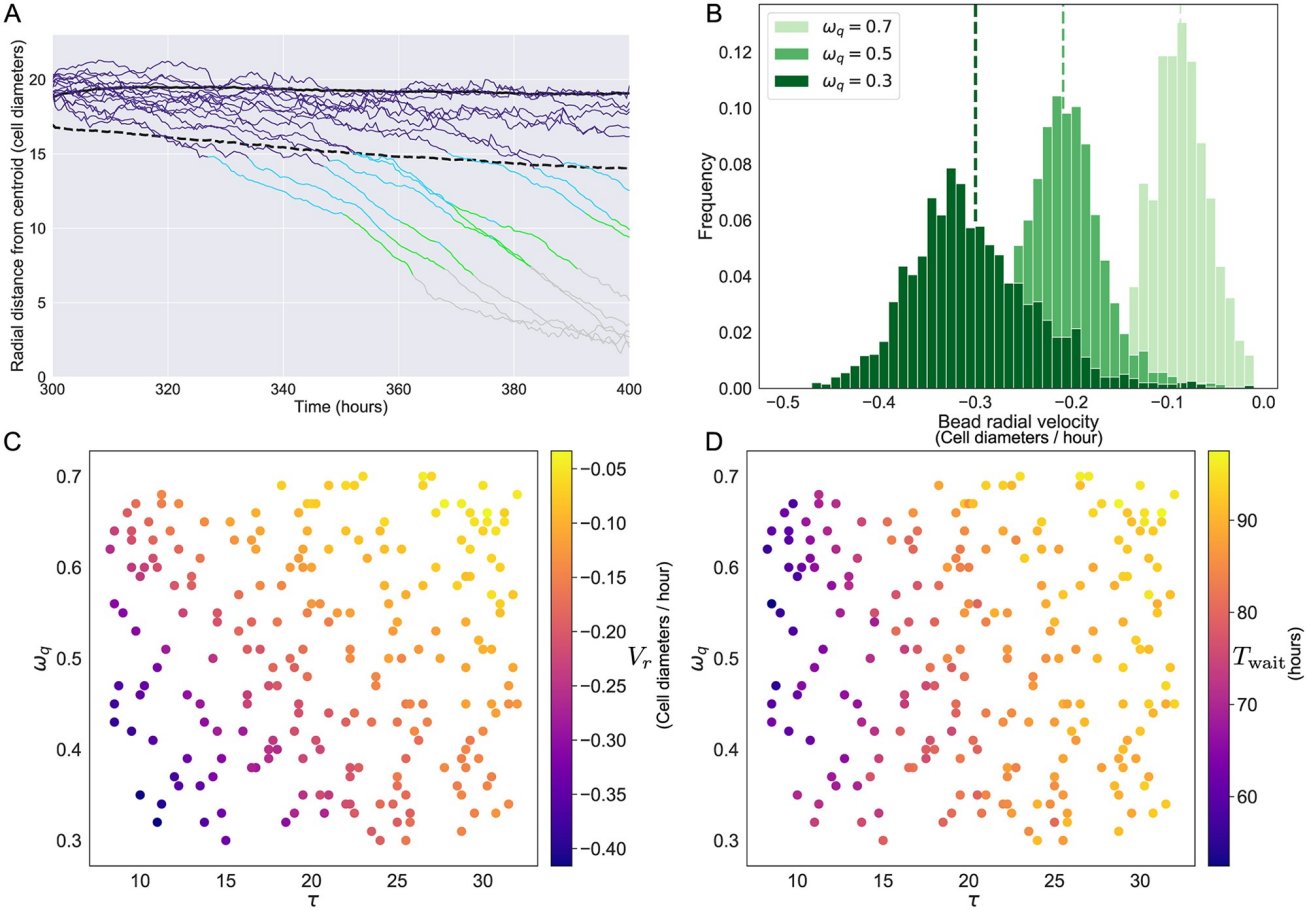
The radial trajectory of individual microbeads correlates with simulation parameters. A: Radial trajectories of 15 beads infiltrating a tumour spheroid (parameters *ω*_q_ = 0.5, *ω*_h_ = 0.3, *τ* = 16, $\tilde{\tau }=8$). Solid black line: approximate radius of tumour spheroid; dashed black line: threshold between regimes based on estimated microbead Brownian motion coefficient *D*_est_ ($\text{Threshold}=0.9\times \text{Spheroidradius}-\sqrt{2D_{\text{est}}t}$, see main text for details). Trajectories are coloured according to the oxygen concentration at the microbead location. We distinguish two phases of infiltration: beads which have not crossed the dashed threshold appear to move by Brownian motion, while those which have crossed the threshold move predominantly by advection. B: Average radial velocity (cell diameters per hour) of infiltrating microbeads within 50 repeats of a representative parameter set for three values of *ω*_q_ (parameters *ω*_h_ = 0.3, *τ* = 16, $\tilde{\tau }=8$). Dashed vertical lines show the mean radial velocity for each distribution. C and D: Plots showing how the average radial velocity *V*_*r*_ and the waiting time *T*_wait_ change as *τ* and *ω*_q_ vary, for fixed *ω*_h_ = 0.3 and $\tilde{\tau }=8$.

Panel A of Fig 5 shows the radial trajectory of 15 randomly selected beads from spheroids simulated with the same parameter set (*ω*_q_ = 0.5, *ω*_h_ = 0.3, *τ* = 16, $\tilde{\tau }=8$). Microbeads are coloured according to the oxygen concentration at their position. Fig 5 shows that microbead trajectories typically have two distinct phases. Initially, microbeads remain in the proliferating rim close to the spheroid boundary where their movement is characterised by random movement generated by tumour cell proliferation. During cell division, the new daughter cell is placed randomly in a neighbourhood of the parent cell. This position may be closer to the spheroid centre than the parent cell, or closer to the spheroid edge. Since the new location is selected randomly, the forces which act on a microbead that is close to a dividing cell due to the presence of the new cell are as likely to push the bead radially inwards or outwards. Since we expect the forces directed outwards to be comparable with those directed inwards over time, we expect radial movement in this regime to be dominated by Brownian motion and neglect movement in the tangential direction in our analysis. On leaving the proliferating region, the microbeads typically move on a linear trajectory towards the spheroid centre. Although the time microbeads remain in the proliferative rim varies, once they enter the central region they appear to move radially inwards at the same velocity. For each bead *i*, we define its *radial infiltration velocity*, $V_{r}^{i}$, to be its average radial velocity from when the bead enters the second regime until it reaches the necrotic core. Similarly, its *waiting time*, $T_{\text{wait}}^{i}$, is the time taken for a microbead to leave the outer proliferating rim and to enter the inner region. We denote the average of these quantities as $V_{r}=\frac{1}{n}\sum_{i}V_{r}^{i}$ and $T_{\text{wait}}=\frac{1}{n}\sum_{i}T_{\text{wait}}^{i}$, where *n* is the number of beads.

We use observations of the microbead velocities to identify the distance from the spheroid boundary at which cell movement transitions from Brownian dominated to advection dominated. The velocities from the first 5 hours of simulation are used to estimate *D*_est_, the estimated diffusion coefficient for Brownian motion. The expected displacement of a particle undergoing 1D Brownian motion for a time *t* is $\sqrt{2D_{\text{est}}t}$, and we use this distance to estimate the location of the threshold separating the two regimes. As this value is close to 0 when *t* is small, we offset this boundary to ensure that microbeads do not cross this threshold during the first timestep. If a spheroid has radius *R* at time *t* after adding microbeads, we estimate that the threshold between regimes is $\text{Threshold}=0.9R-\sqrt{2D_{\text{est}}t}$. This estimated boundary is shown as a dashed black line in Panel A of Fig 5.

Panel B of Fig 5 shows distributions of $V_{r}{}^{i}$ for three different values of *ω*_q_ (*ω*_h_ = 0.3, *τ* = 16, ${\tilde{\tau }}_{i}=8$, 50 simulation repetitions). Each distribution has a clear peak which indicates the presence of a cellular flow of tumour cells from the proliferative rim of the spheroid towards the dying cells in the necrotic core with an approximately constant radial velocity. The vertical dashed lines correspond to *V*_*r*_ for each parameter set. Panel C of Fig 5 shows how *V*_*r*_ varies with *τ* and *ω*_q_, for randomly sampled combinations of the two parameters with all other parameters fixed. Beads exhibit a faster inward radial velocity when the average cell cycle is faster, and also when the threshold for quiescence is low (generating a wider rim of proliferating cells).

Panel D of Fig 5 shows how varying *ω*_q_ and *τ* affects *T*_wait_, the mean time that a microbead spends in the random motion dominated regime. Reducing *τ* lowers *T*_wait_, indicating that bead movement across this part of the spheroid is driven by cell proliferation. Reducing *ω*_q_ also increased the time spent near the spheroid edge for small *τ*, although this effect is less prominent as *τ* increases.

### Inferring spheroid composition and parameters from microbead trajectories

**Fig 6:**
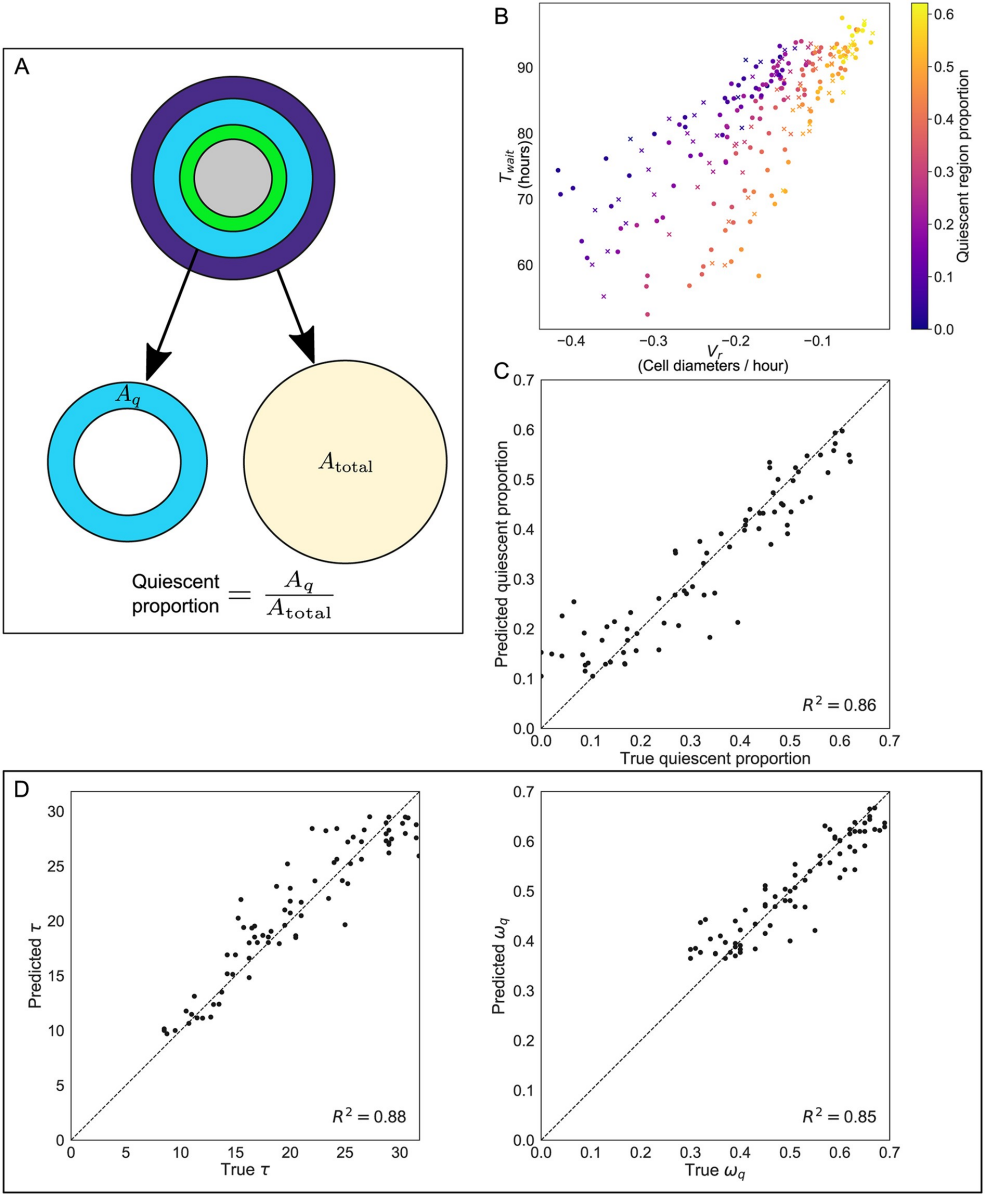
Observations of *T*_wait_ and *V*_*r*_ can be used to predict spheroid composition and simulation parameters. A: Schematic showing how the composition of the spheroid was defined in order to make predictions. The proportion of the spheroid which was quiescent is defined as the area of the quiescent region, *A*_*q*_, divided by the total spheroid area *A*_total_. (Comparable results are obtained when defining the quiescent proportion of the spheroid as the width of the quiescent annulus divided by the total spheroid radius.) B: Proportion of the spheroid radius accounted for by the quiescent compartment for simulations with randomly selected *ω*_q_ and *τ* (with fixed *ω*_h_ = 0.3 and $\tilde{\tau }=8$). Approximately two-thirds of the simulations (100 out of 148, marked as circles) were used to train a *k*-nearest neighbours classifier to predict the composition of spheroids in the remaining simulations (48 out of 148, marked as crosses). C: Results of using 10-nearest neighbours classification to predict the proportion of each tumour spheroid which was quiescent based on observed values of *T*_wait_ and *V*_*r*_ for microbeads in each simulation. D: Comparison of parameter values for (left) *τ* and (right) *ω*_q_ based on 10-nearest neighbours classification with the values used to generate the synthetic data.

Fig 4 shows that spheroid composition is correlated with *τ* and *ω*_q_, while Fig 5 shows that the same parameters are correlated with *T*_wait_ and *V*_*r*_. Motivated by these results, we now demonstrate that observations of *T*_wait_ and *V*_*r*_ can be used to identify features of spheroid composition. In panel A of Fig 6 we define the quiescent proportion of the spheroid as the area of the tumour spheroid (*A*_total_) occupied by the quiescent compartment (*A*_*q*_). We use a 2D metric, as our simulations are in 2D; a similar 1D metric based on the relative widths of the tumour spheroid and quiescent compartment produces similar results. Panels B and C illustrate that the quiescent proportion can be estimated accurately by observing *T*_wait_ and *V*_*r*_ for a given simulation.

In panel B of Fig 6 we present results from simulations involving a range of spheroids generated using randomly selected values of *ω*_q_ and *τ*. The markers are coloured according to the quiescent proportion of each simulated spheroid, and are scattered according to the values of *T*_wait_ and *V*_*r*_ observed in each simulation. Spheroids with similarly sized quiescent regions are found close together, suggesting that measurements of individual microbead velocities and waiting times could be used to predict the composition of a tumour spheroid. The same effect can be observed for spheroids with similarly sized proliferating, hypoxic and necrotic regions (see S6 Appendix: Predicting spheroid composition from microbead observations).

We applied a *k*-nearest neighbours classifier [61] (with *k* = 10) to predict the composition of the spheroid based on the observed values of *T*_wait_ and *V*_*r*_. The simulations were split into two groups to use as training and testing data for the classifier (100 data points for training, 48 data points for prediction, shown as circles and crosses respectively in Fig 6, panel B). Panel C of Fig 6 shows that the predictions of this classifier are accurate, with a high *R*^2^ value (*R*^2^ = 0.86) indicating that the predictions are well correlated with the true quiescent proportions of the simulated spheroids in the validation set. Similarly, it is possible to predict the size of the proliferating, hypoxic and necrotic regions (see S6 Appendix: Predicting spheroid composition from microbead observations).

As well as predicting the spheroid composition, *T*_wait_ and *V*_*r*_ can also be used to infer parameters associated with spheroid growth. Panel D of Fig 6 shows scatterplots in which the true values of *τ* and *ω*_q_ for simulations in the validation dataset are compared with the predictions from the nearest neighbours classifier. Observing the trajectories of individual beads allows accurate prediction of the average cell cycle length (*R*^2^ = 0.88) and oxygen threshold for quiescence (*R*^2^ = 0.85) of simulated tumour cells.

## Discussion

We have developed a hybrid, off-lattice ABM for oxygen-limited spheroid growth. Our model reproduces the sigmoidal growth dynamics seen in *in vitro* spheroids as well as their spatial structure, consisting of an outer rim of proliferating cells and a central necrotic core (Fig 2). Our simulations reveal that it is possible to generate synthetic spheroids with similar growth dynamics but different spatial compositions of proliferating, quiescent, hypoxic and necrotic cells (Fig 4). These changes in spheroid composition are driven by variations in cell-scale behaviours such as the average length of the cell cycle or the oxygen thresholds at which cells are affected by hypoxia.

The model describes the passive infiltration of microbeads into tumour spheroids and reproduces the distributions of microbeads described by Dorie *et al*. [46] (Fig 3). It also reproduces the dynamics of infiltrating ^3^H-labelled cells described in [46], and can explain differences in the spatial distributions of the microbeads and ^3^H-labelled cells without invoking additional mechanisms such as chemotaxis (see Supporting Information, S5 Appendix: Replication of ^3^H-labelled cells experiments). In contrast to previous continuum models describing this data, this model can distinguish the trajectories of individual microbeads. This allows us to derive experimentally measurable properties of microbead infiltration, *T*_wait_ and *V*_*r*_, which permit accurate prediction of both model parameters and the spheroid composition. *T*_wait_ describes the average length of time which a microbead spends close to the spheroid edge, and correlates with the average length of the cell cycle, *τ* (Fig 5). *V*_*r*_ is the speed at which microbeads move radially inwards once they have moved sufficiently far from the spheroid boundary to enter the advection dominated regime inside the spheroid, and correlates with both *τ* and the oxygen threshold at which cells become quiescent, *ω*_q_ (Fig 5). Combinations of *V*_*r*_ and *T*_wait_ can be used to accurately predict the values of *τ* and *ω*_q_ used to simulate a spheroid (Fig 6), as well as the proportion of the spheroid consisting of proliferating, quiescent, hypoxic or necrotic cells (Fig 6 and Supporting Information, S6 Appendix: Predicting spheroid composition from microbead observations).

The model enables detailed simulation of the forces acting on microbeads during passive migration. In common with other agent-based models, it includes a large number of parameters. The results presented here were obtained by fixing many of the parameters at values specified in S7 Appendix: Table of parameters. While some of these parameters have been informed by the literature, this model has identified those that should be informed by further experiments to identify the relationship between spheroid simulations and *in vitro* spheroids. We restrict our analysis to 2D tumour spheroids, for several reasons. A single realisation of a 2D simulation may take several hours to run on one core of a desktop PC; by contrast a 3D simulation of a spheroid containing tens of thousands of cells would take several days to complete. This computational effort would make it prohibitive to conduct in 3D the parameter sensitivity analyses performed in this paper, particularly as multiple simulations are required for each parameter set. Further, the 2D simulations described here are sufficiently detailed to identify qualitative differences in passive migration patterns as parameters relating to tumour cell proliferation and death are varied. Identifying the relationships between models of tumour spheroid growth implemented in 2D or 3D, or implemented within different software frameworks, remains an open problem.

We note that in the necrotic core of some spheroids observed in *in vitro* experiments, loss of volume in dead cells due to fluid leakage can cause “cracking”, or fluid filled voids, when coupled with intercellular adhesion [62, 63]. While this behaviour has been reported in existing agent-based models of tumour spheroids [32, 37], we note that for our simulations this effect is only observed when the magnitude of the surface tension force *β* is close to zero. As predicted by Landman and Please [64], spheroids featuring this “void” effect frequently produce travelling wave solutions which do not reach a steady state when this force resisting spheroid growth is sufficiently small.

Extensions of our model could include the response of tumour spheroids to treatments such as radiotherapy or chemotherapy which are most effective at targetting proliferating cells. Our agent-based model could be used to examine the role that tumour composition plays during these therapies, as previous continuum models predict that treatment response is highly dependent on the internal composition of tumours [12]. Advection has also been identified as being of importance in models of drug treatment efficiency (e.g., [49, 50]). Advection-diffusion equations have also been used to model the growth of vascular tumours [65], and the development of tumour metastasis [66].

This model could also be extended to investigate tumour-immune interactions in which immune cells, such as macrophages, infiltrate into clusters of tumour cells. Previous continuum models of macrophage infiltration into tumour spheroids and avascular tumours emphasise the role of chemotaxis [67–71] or paracrine signalling [72, 73]. However, to date the influence of passive migration on the infiltration of immune cells into spheroids and avascular tumours has not been considered. Much focus has been placed on the impact that immune cells have on tumour cells, but the impact of solid tumours on the spatial distributions of immune cells has attracted less attention.

In future work we could also extend the model to account for other metabolites and waste products which impact tumour spheroid growth, such as glucose, lactate and pH levels [21]. These model extensions could be used to simulate tumour responses to metabolic inhibitors or treatments that are affected by pH.

We have used an agent-based framework to simulate passive migration into tumour spheroids. This migration is caused by the collective movement of tumour cells, which is itself a consequence of the spatial composition of a tumour spheroid. This model shows that passive migration is strongly influenced by spheroid composition, and shows how microbead infiltration experiments similar to those conducted by Dorie *et al*. [46] could be used to deduce this composition.

## Supporting information

S1 Appendix Spring force magnitude. (PDF)

S2 Appendix Algorithm for updating the cell cycle. (PDF)

S3 Appendix Non-dimensionalisation of oxygen equation. (PDF)

S4 Appendix Microbead infiltration histograms for other parameter combinations. (PDF)

S5 Appendix Replication of ^3^H-labelled cells experiments. (PDF)

S6 Appendix Predicting spheroid composition from microbead observations. (PDF)

S7 Appendix Table of parameters. (PDF)

S8 Appendix Impact of initial spheroid radius on steady state dynamics. (PDF)

S9 Appendix schematic of forces acting on cells. (PDF)

## References

[1] Dagogo-Jack I, Shaw AT. Tumour heterogeneity and resistance to cancer therapies. Nature Reviews Clinical Oncology. 2018;15(2):81–94. 10.1038/nrclinonc.2017.166 29115304

[2] Weiswald LB, Bellet D, Dangles-Marie V. Spherical cancer models in tumor biology. Neoplasia. 2015;17(1):1–15. 10.1016/j.neo.2014.12.004 25622895 PMC4309685

[3] Folkman J, Hochberg M. Self-regulation of growth in three dimensions. The Journal of Experimental Medicine. 1973;138(4):745–753. 10.1084/jem.138.4.745 4744009 PMC2180571

[4] Sutherland RM. Cell and environment interactions in tumor microregions: The multicell spheroid model. Science. 1988;240(4849):177–184. 10.1126/science.2451290 2451290

[5] Proskuryakov SY, Konoplyannikov AG, Gabai VL. Necrosis: A specific form of programmed cell death? Experimental Cell Research. 2003;283(1):1–16. 12565815 10.1016/s0014-4827(02)00027-7

[6] Guillaume L, Rigal L, Fehrenbach J, Severac C, Ducommun B, Lobjois V. Characterization of the physical properties of tumor-derived spheroids reveals critical insights for pre-clinical studies. Scientific Reports. 2019;9:6597 10.1038/s41598-019-43090-0 31036886 PMC6488646

[7] Karolak A, Markov DA, McCawley LJ, Rejniak KA. Towards personalized computational oncology: from spatial models of tumour spheroids, to organoids, to tissues. Journal of the Royal Society Interface. 2018;15(20170703) 10.1098/rsif.2017.0703 PMC5805971 29367239

[8] Stéphanou A, Ballet P, Powathil G. Hybrid data-based modelling in oncology: successes, challenges and hopes. Mathematical Modelling of Natural Phenomena. 2020;15(21)

[9] Karolak A, Poonja S, Rejniak KA. Morphophenotypic classification of tumor organoids as an indicator of drug exposure and penetration potential. PLoS Computational Biology. 2019;15(7) 10.1371/journal.pcbi.1007214 31310602 PMC6660094

[10] Montes-Olivas S, Marucci L, Homer M. Mathematical Models of Organoid Cultures. Frontiers in Genetics. 2019;10(873) 10.3389/fgene.2019.00873 31592020 PMC6761251

[11] Brüningk S, Powathil G, Ziegenhein P, Ijaz J, Rivens I, Nill S, Chaplain M, Oelfke U, ter Haar G. Combining radiation with hyperthermia: A multiscale model informed by in vitro experiments. Journal of the Royal Society Interface. 2018;15:20170681 10.1098/rsif.2017.0681 PMC5805969 29343635

[12] Lewin TD, Maini PK, Moros EG, Enderling H, Byrne HM. The Evolution of Tumour Composition During Fractionated Radiotherapy: Implications for Outcome. Bulletin of Mathematical Biology. 2018;80;1207–1235 10.1007/s11538-018-0391-9 29488054

[13] Spratt JA, von Fournier D, Spratt JS, Weber EE. Decelerating growth and human breast cancer. Cancer. 1993;71(6):2013–2019. 8443753 10.1002/1097-0142(19930315)71:6<2013::aid-cncr2820710615>3.0.co;2-v

[14] Steel GG, Lamerton LF. The growth rate of human tumours. British Journal of Cancer. 1966;20(1):74–86. 10.1038/bjc.1966.9 5327764 PMC2008056

[15] Araujo RP, McElwain DLS. A history of the study of solid tumour growth: The contribution of mathematical modelling. Bulletin of Mathematical Biology. 2004;66(5):1039–1091. 10.1016/j.bulm.2003.11.002 15294418

[16] Greenspan HP. Models for the growth of a solid tumor by diffusion. Studies in Applied Mathematics. 1972;51(4):317–340. 10.1002/sapm1972514317

[17] Greenspan HP. On the growth and stability of cell cultures and solid tumors. Journal of Theoretical Biology. 1976;56(1):229–242. 10.1016/S0022-5193(76)80054-9 1263527

[18] Ward JP, King JR. Mathematical modelling of avascular-tumour growth. IMA Journal of Mathematics Applied in Medicine and Biology. 1997;14(1):39–69. 10.1093/imammb/14.1.39 9080687

[19] Byrne HM, Chaplain MAJ. Necrosis and apoptosis: Distinct cell loss mechanisms in a mathematical model of avascular tumour growth. Journal of Theoretical Medicine. 1998;1(3):223–235. 10.1080/10273669808833021

[20] Ward JP, King JR. Mathematical modelling of avascular-tumour growth. II: Modelling growth saturation. IMA journal of mathematics applied in medicine and biology. 1999;16(2):171–211. 10.1093/imammb/16.2.171 10399312

[21] Jagiella N, Müller B, Müller M, Vignon-Clementel IE, Drasdo D. Inferring Growth Control Mechanisms in Growing Multi-cellular Spheroids of NSCLC Cells from Spatial-Temporal Image Data. PLoS Computational Biology. 2016;12(2) 10.1371/journal.pcbi.1004412 26866479 PMC4750943

[22] Aleksandrova AV, Pulkova NP, Gerasimenko TN, Anisimov NY, Tonevitskaya SA, Sakharov DA. Mathematical and Experimental Model of Oxygen Diffusion for HepaRG Cell Spheroids. Bulletin of Experimental Biology and Medicine. 2015;160(12):836–840. 10.1007/s10517-016-3326-1 27165074

[23] Michel T, Fehrenbach J, Lobjois V, Laurent J, Gomes A, Colin T, Poignard C. Mathematical modeling of the proliferation gradient in multicellular tumor spheroids. Journal of Theoretical Biology. 2018;458:133–147. 10.1016/j.jtbi.2018.08.031 30145131

[24] McMurtrey RJ. Analytic models of oxygen and nutrient diffusion, metabolism dynamics, and architecture optimization in three-dimensional tissue constructs with applications and insights in cerebral organoids. Tissue Engineering: Part C. 2016;22(3);221–248. 10.1089/ten.tec.2015.0375 PMC5029285 26650970

[25] Caraguel F, Lesart A-C, Estève F, van der Sanden B, Stéphanou A. Towards the Design of a Patient-Specific Virtual Tumour. Computational and Mathematical Methods in Medicine. 2016;7851789 10.1155/2016/7851789 28096895 PMC5206790

[26] Alarcón T, Byrne HM, Maini PK. A cellular automaton model for tumour growth in inhomogeneous environment. Journal of Theoretical Biology. 2003;225(2):257–274. 10.1016/S0022-5193(03)00244-3 14575659

[27] Piotrowska MJ, Angus SD. A quantitative cellular automaton model of in vitro multicellular spheroid tumour growth. Journal of Theoretical Biology. 2009;258:165–178. 10.1016/j.jtbi.2009.02.008 19248794

[28] Graner F, Glazier JA. Simulation of biological cell sorting using a two-dimensional extended Potts model. Physical Review Letters. 1992;69(13):2013–2017. 10.1103/PhysRevLett.69.2013 10046374

[29] Poplawski NJ, Shirinifard A, Agero U, Gens JS, Swat M, Glazier JA. Front instabilities and invasiveness of simulated 3D avascular tumors. PLoS ONE. 2010;5(5):e10641 10.1371/journal.pone.0010641 20520818 PMC2877086

[30] Shirinifard A, Gens JS, Zaitlen BL, Popławski NJ, Swat M, Glazier JA. 3D multi-cell simulation of tumor growth and angiogenesis. PLoS ONE. 2009;4(10):e7190 10.1371/journal.pone.0007190 19834621 PMC2760204

[31] Osborne JM, Fletcher AG, Pitt-Francis JM, Maini PK, Gavaghan DJ. Comparing individual-based approaches to modelling the self-organization of multicellular tissues. PLoS Computational Biology. 2017;13(2):1–34. 10.1371/journal.pcbi.1005387 PMC5330541 28192427

[32] Drasdo D, Höhme S. A single-cell-based model of tumor growth in vitro: Monolayers and spheroids. Physical Biology. 2005;2(3):133–147. 10.1088/1478-3975/2/3/001 16224119

[33] Meineke FA, Potten CS, Loeffler M. Cell migration and organization in the intestinal crypt using a lattice-free model. Cell Proliferation. 2001;34(4):253–266. 10.1046/j.0960-7722.2001.00216.x 11529883 PMC6495866

[34] Cleri F. Agent-based model of multicellular tumor spheroid evolution including cell metabolism. The European Physical Journal E. 2019;42(8). 10.1140/epje/i2019-11878-7 31456065

[35] Mirams GR, Arthurs CJ, Bernabeu MO, Bordas R, Cooper J, Corrias A, et al Chaste: An open source C++ library for computational physiology and biology. PLoS Computational Biology. 2013;9(3). 10.1371/journal.pcbi.1002970 23516352 PMC3597547

[36] Pitt-Francis J, Pathmanathan P, Bernabeu MO, Bordas R, Cooper J, Fletcher AG, et al Chaste: A test-driven approach to software development for biological modelling. Computer Physics Communications. 2009;180(12):2452–2471. 10.1016/j.cpc.2009.07.019

[37] Ghaffarizadeh A, Heiland R, Friedman SH, Shannon M. PhysiCell: An open source physics-based cell simulator for 3-D multicellular systems. PLoS Computational Biology. 2018;14(2):1–34. 10.1371/journal.pcbi.1005991 PMC5841829 29474446

[38] Ghaffarizadeh A, Friedman SH, MacKlin P. BioFVM: An efficient, parallelized diffusive transport solver for 3-D biological simulations. Bioinformatics. 2016;32(8):1256–1258. 10.1093/bioinformatics/btv730 26656933 PMC4824128

[39] Starruß J, De Back W, Brusch L, Deutsch A. Morpheus: A user-friendly modeling environment for multiscale and multicellular systems biology. Bioinformatics. 2014;30(9):1331–1332. 10.1093/bioinformatics/btt772 24443380 PMC3998129

[40] Swat MH, Thomas GL, Belmonte JM, Shirinifard A, Hmeljak D, Glazier JA. Multi-scale modeling of tissues using CompuCell3D. Methods in Cell Biology. 2012;110:325–366. 10.1016/B978-0-12-388403-9.00013-8 22482955 PMC3612985

[41] Hoehme S, Drasdo D. A cell-based simulation software for multi-cellular systems. Bioinformatics. 2010;26(20):2641–2642. 10.1093/bioinformatics/btq437 20709692 PMC2951083

[42] Kang S, Kahan S, McDermott J, Flann N, Shmulevich I. Biocellion: Accelerating computer simulation of multicellular biological system models. Bioinformatics. 2014;30(21):3101–3108. 10.1093/bioinformatics/btu498 25064572 PMC4609016

[43] Bravo R, Baratchart E, West J, Schenck RO, Miller AK, Gallaher J, et al Hybrid Automata Library: A modular platform for efficient hybrid modeling with real-time visualization. Preprint bioRxiv. 2018. 10.1371/journal.pcbi.1007635 PMC7105119 32155140

[44] Cytowski M, Szymanska Z. Large-scale parallel simulations of 3D cell colony dynamics: The cellular environment. Computing in Science and Engineering. 2015;17(5):44–48. 10.1109/MCSE.2015.66

[45] Helmlinger G, Netti Pa, Lichtenbeld HC, Melder RJ, Jain RK. Solid stress inhibits the growth of multicellular tumor spheroids. Nature biotechnology. 1997;15(8):778–783. 10.1038/nbt0897-778 9255794

[46] Dorie MJ, Kallman RF, Rapacchietta DF, Van Antwerp D, Huang YR. Migration and internalization of cells and polystyrene microspheres in tumor cell spheroids. Experimental Cell Research. 1982;141(1):201–209. 10.1016/0014-4827(82)90082-9 7117414

[47] Dorie MJ, Kallman RF, Coyne MA. Effect of cytochalasin B, nocodazole and irradiation on migration and internalization of cells and microspheres in tumor cell spheroids. Experimental Cell Research. 1986;166(2):370–378. 10.1016/0014-4827(86)90483-0 3743661

[48] Delarue M, Montel F, Caen O, Elgeti J, Siaugue J-M, Vignjevic D, Prost J, Joanny J-F, Cappello G. Mechanical control of cell flow in multicellular spheroids. Physical Review Letters. 2013;110(13) 10.1103/PhysRevLett.110.138103 23581378

[49] Jain RK. Transport of Molecules in the Tumor Interstitium: A Review. Cancer Research. 1987;47(12);3039–3051 https://cancerres.aacrjournals.org/content/47/12/3039. 3555767

[50] Rejniak KA, Estrella V, Chen T, Cohen AS, Lloyd MC, Morse DL. The role of tumor tissue architecture in treatment penetration and efficacy: an integrative study. Frontiers in Oncology. 2013;3(111) 10.3389/fonc.2013.00111 PMC3650652 23717812

[51] McElwain DLS, Pettet GJ. Cell migration in multicell spheroids: Swimming against the tide. Bulletin of Mathematical Biology. 1993;55(3):655–674. 8364422 10.1007/BF02460655

[52] Thompson KE, Byrne HM. Modelling the internalization of labelled cells in tumour spheroids. Bulletin of Mathematical Biology. 1999;61(4):601–623. 10.1006/bulm.1999.0089 17883217

[53] Pettet GJ, Please CP, Tindall MJ, Mcelwain DLS. The migration of cells in multicell tumor spheroids. Bulletin of Mathematical Biology. 2001;63(2):231–257. 10.1006/bulm.2000.0217 11276525

[54] Harvey DG, Fletcher AG, Osborne JM, Pitt-Francis J. A parallel implementation of an off-lattice individual-based model of multicellular populations. Computer Physics Communications. 2015;192:130–137. 10.1016/j.cpc.2015.03.005

[55] Greijer AE, Van Der Wall E. The role of hypoxia inducible factor 1 (HIF-1) in hypoxia induced apoptosis. Journal of Clinical Pathology. 2004;57(10):1009–1014. 10.1136/jcp.2003.015032 15452150 PMC1770458

[56] Perfahl H, Hughes BD, Alarcón T, Maini PK, Lloyd MC, Reuss M, Byrne HM. 3D hybrid modelling of vascular network formation. Journal of Theoretical Biology. 2017;414:254–268. 10.1016/j.jtbi.2016.11.013 27890575

[57] Drasdo D, Loeffler M. Individual-based models to growth and folding in one-layered tissues: Intestinal crypts and early development. Nonlinear Analysis. 2001;47:245–256. 10.1016/S0362-546X(01)00173-0

[58] Usami Y, Ishida K, Sato S, Kishino M, Kiryu M, Ogawa Y, et al Intercellular adhesion molecule-1 (ICAM-1) expression correlates with oral cancer progression and induces macrophage/cancer cell adhesion. International Journal of Cancer. 2013;133(3):568–578. 10.1002/ijc.28066 23364881

[59] Edelsbrunner H, Kirkpatrick DG, Seidel R. On the shape of a set of points. IEEE Transactions on Information Theory. 1983;29(4):551–559. 10.1109/TIT.1983.1056714

[60] Grimes DR, Kelly C, Bloch K, Partridge M A method for estimating the oxygen consumption rate in multicellular tumour spheroids. Journal of the Royal Society Interface. 2014;11:20131124 10.1098/rsif.2013.1124 PMC3899881 24430128

[61] Altman NS. An introduction to kernel and nearest-neighbor nonparametric regression. American Statistician. 1992;46(3):175–185. 10.1080/00031305.1992.10475879

[62] Ma HL, Jiang Q, Han S, Wu Y, Tomshine JC, Wang D, et al Multicellular tumor spheroids as an in vivo-like tumor model for three-dimensional imaging of chemotherapeutic and nano material cellular penetration. Molecular Imaging. 2012;11(6):487–498. 10.2310/7290.2012.00012 23084249

[63] Ghosh S, Joshi MB, Ivanov D, Feder-Mengus C, Spagnoli GC, Martin I, et al Use of multicellular tumor spheroids to dissect endothelial cell-tumor cell interactions: A role for T-cadherin in tumor angiogenesis. FEBS Letters. 2007;581(23):4523–4528. 10.1016/j.febslet.2007.08.038 17765896

[64] Landman KA, Please CP. Tumour dynamics and necrosis: Surface tension and stability. IMA Journal of Mathematics Applied in Medicine and Biology. 2001;18(2):131–158. 10.1093/imammb/18.2.131 11453466

[65] De Angelis E, Preziosi L. Advection-diffusion models for solid tumour evolution in vivo and related free boundary problem. Mathematical Models and Methods in Applied Sciences. 2000;10(3);379–407 10.1142/S0218202500000239

[66] Baratchart E, Benzekry S, Bikfalvi A, Colin T, Cooley LS, Pineau R, Ribot EJ, Saut O, Souleyreau W. Computational Modelling of Metastasis Development in Renal Cell Carcinoma. PLoS Computational Biology. 2015;11(11);e1004626 10.1371/journal.pcbi.1004626 26599078 PMC4658171

[67] Owen MR, Sherratt JA. Modelling the macrophage invasion of tumours: Effects on growth and composition. IMA Journal of Mathematics Applied in Medicine and Biology. 1998;15(2):165–185. 10.1093/imammb/15.2.165 9661282

[68] Owen MR, Sherratt JA. Pattern formation and spatiotemporal irregularity in a model for macrophage-tumour interactions. Journal of theoretical biology. 1997;189(1):63–80. 10.1006/jtbi.1997.0494 9398504

[69] Owen MR, Sherratt JA. Mathematical modelling of macrophage dynamics in tumours. Mathematical Models and Methods in Applied Sciences. 1999;9(4):513–539. 10.1142/S0218202599000270

[70] Kelly CE, Leek RD, Byrne HM, Cox SM, Harris AL, Lewis CE. Modelling macrophage infiltration into avascular tumours. Journal of Theoretical Medicine. 2002;4(1):21–38. 10.1080/10273660290015242

[71] Webb SD, Owen MR, Byrne HM, Murdoch C, Lewis CE. Macrophage-based anti-cancer therapy: Modelling different modes of tumour targeting. Bulletin of Mathematical Biology. 2007;69(5):1747–1776. 10.1007/s11538-006-9189-2 17333419

[72] Knútsdóttir H, Pálsson E, Edelstein-Keshet L. Mathematical model of macrophage-facilitated breast cancer cells invasion. Journal of Theoretical Biology. 2014;357:184–199. 10.1016/j.jtbi.2014.04.031 24810842

[73] Knútsdóttir H, Condeelis JS, Pálsson E. 3-D individual cell based computational modeling of tumor cell–macrophage paracrine signaling mediated by EGF and CSF-1 gradients. Integrative Biology. 2016;8(1):104–119. 10.1039/c5ib00201j 26686751 PMC5013833

