# Supplementary material for: Mathematical modelling reveals cellular dynamics within tumour spheroids
Source: PLoS Comput Biol. 2020 Aug 18;16(8):e1007961. doi: 10.1371/journal.pcbi.1007961 (PMC7455028; doi:10.1371/journal.pcbi.1007961)
Supplement: S1 Appendix — (PDF) [file pcbi.1007961.s001.pdf]

Supporting information

For: **Mathematical modelling reveals cellular dynamics within tumour spheroids**
by Joshua A. Bull, Franziska Mech, Tom Quaiser, Sarah L. Waters, Helen M. Byrne

S1 Appendix: Spring force magnitude Fig A shows the magnitude of the spring force described in Eq (8) for the default parameter values used in our simulations. The form of the equation is chosen such that as neighbouring cells approach each other the force between them gradually increases, modelling the increasing number of bonds holding the cells together as the surface area between them increases. If the cells are forced too close together then they will attempt to repel each other, described in the graph as a force with negative magnitude. This force becomes stronger as cell centres are forced together, providing a model of volume exclusion. The cells will attempt to reach an equilibrium distance which models the optimum balance between cell-cell adhesion and volume exclusion.

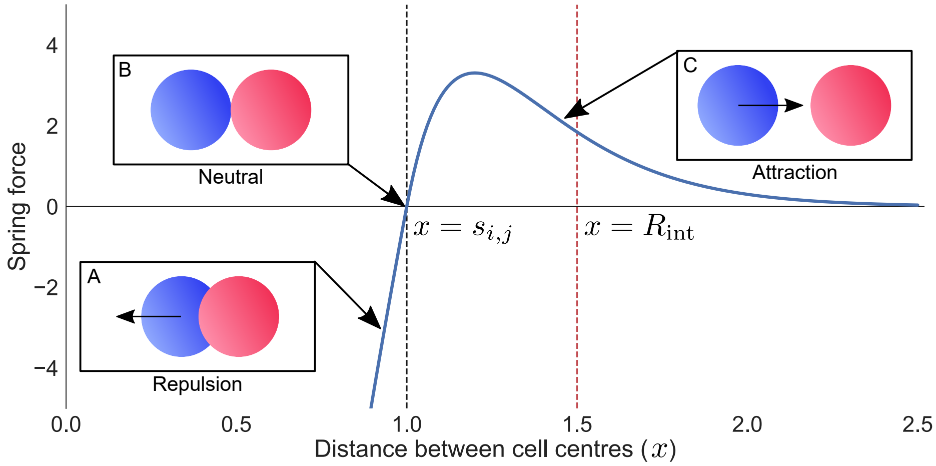

Fig A. Schematic showing the magnitude of the spring force between neighbouring cells as the distance between them varies.
This schematic shows how the magnitude and sign of the spring forces acting on two cells depends on x, the distance between their centres, for fixed values of the model parameters (see Fig 1 and Eq (8)). The vertical black dashed line indicates where $x = s_{i,j}$ and the corresponding spring force vanishes; the vertical red dashed line indicates where $x = R_{\text{int}}$. A: If the distance between cells i and j is less than $s_{i,j}$, then they experience a repulsive force which increases exponentially as their separation decreases. B: When $x = s_{i,j}$, the spring force is zero. C: If the distance between cells is greater than $s_{i,j}$, then they are attracted towards one another. Parameter values: $s_{i,j} = 1$, $R_{\text{int}} = 1.5$, $\mu = 45$, $\lambda = 5$ (see Eq (8) for details).
