# Supplementary material for: Mathematical modelling reveals cellular dynamics within tumour spheroids
Source: PLoS Comput Biol. 2020 Aug 18;16(8):e1007961. doi: 10.1371/journal.pcbi.1007961 (PMC7455028; doi:10.1371/journal.pcbi.1007961)
Supplement: S2 Appendix — (PDF) [file pcbi.1007961.s002.pdf]

Supporting information

For: **Mathematical modelling reveals cellular dynamics within tumour spheroids**
by Joshua A. Bull, Franziska Mech, Tom Quaiser, Sarah L. Waters, Helen M. Byrne

S2 Appendix: Algorithm for updating the cell cycle Algorithm 1 shows an outline of the algorithm used to update the spring lengths and cell cycles of each cell at each timestep.

```
Input: All cells, viable or necrotic
for All cells do
    if Cell is alive then
        if ω_q < ω ≤ 1 then
            // Cell is proliferative
            // Move cell through cell cycle by one timestep
            Set T_i = T_i + dt;
            // Ensure hypoxia timer is unset
            Set T̃_i = 0;
            // If cell is less than one hour old, increase the cell
               radius
            if T_i < 1 then
                Set s_i = s_i + R_Cell dt;
            end
            // If cell is at end of cell cycle, proliferate
            if T_i = τ_i then
                Choose random location within R_int of cell i;
                Place daughter cell j in selected location;
                Set s_i = R_Cell / 2;
                Set s_j = R_Cell / 2;
                Set T_i = 0 for cells i and j;
                Choose new cell cycle durations τ_i for cells i and j;
            end
        else if ω_h < ω ≤ ω_q then
            // Cell is quiescent
            // Ensure hypoxia timer is unset
            Set T̃_i = 0;
        else if ω ≤ ω_h then
            // Cell is hypoxic
            // Increment hypoxia timer by one timestep
            Set T̃_i = T̃_i + dt;
            // Check for cell death
            if T̃_i = τ̃_i then
                Mark cell as dead;
            end
        end
    else
        // Cell is necrotic
        // Reduce necrotic cell radius linearly over τ̄ hours to
           model decay
        Set s_i = s_i − R_Cell dt / τ̄;
        if s_i = 0 then
            Remove cell from simulation;
        end
    end
end
```

Algorithm 1: Pseudocode outlining the procedure used to update the cell cycle.
