# Supplementary material for: Mathematical modelling reveals cellular dynamics within tumour spheroids
Source: PLoS Comput Biol. 2020 Aug 18;16(8):e1007961. doi: 10.1371/journal.pcbi.1007961 (PMC7455028; doi:10.1371/journal.pcbi.1007961)
Supplement: S3 Appendix — (PDF) [file pcbi.1007961.s003.pdf]

Supporting information

For: **Mathematical modelling reveals cellular dynamics within tumour spheroids**
by Joshua A. Bull, Franziska Mech, Tom Quaiser, Sarah L. Waters, Helen M. Byrne

S3 Appendix: Non-dimensionalisation of oxygen equation The dimensional equation governing the spatio-temporal evolution of the oxygen concentration is:

$$\frac{\partial \omega}{\partial t} = D_\omega \nabla^2 \omega - \kappa \omega \sum_i \delta(\mathbf{x} - \mathbf{x}_i). \tag{S1}$$

where $\mathbf{x}_i$ is the location of cell i, the parameter κ controls the oxygen consumption rate of tumour cells and the parameter D_ω is the diffusion coefficient of oxygen. $\delta(\mathbf{x} - \mathbf{x}_i)$ is a delta function which equals 1 when the argument is zero, and is otherwise equal to zero. We rescale Equation (S1) using the following scales:

$$\tilde{\omega} = \frac{\omega}{\omega_0}; \qquad \tilde{t} = \frac{t}{t_0}; \qquad \tilde{x} = \frac{x}{x_0}; \qquad \tilde{y} = \frac{y}{x_0} \tag{S2}$$

to obtain a non-dimensional form of Equation (S1):

$$\frac{\partial \tilde{\omega}}{\partial \tilde{t}} = \tilde{D_\omega} \nabla^2 \tilde{\omega} - \tilde{\kappa} \tilde{\omega} \sum_i \delta(x_0(\tilde{\mathbf{x}} - \tilde{\mathbf{x}}_i)). \tag{S3}$$

where

$$\tilde{D_\omega} = \frac{D_\omega t_0}{x_0^2} \qquad \text{and} \qquad \tilde{\kappa} = \kappa t_0. \tag{S4}$$

We choose $t_0 = 1$ hour as the timescale and $x_0 = 1$ cell diameter as the lengthscale. Dropping the tildes for convenience, the non-dimensional equation describing the spatio-temporal evolution of the oxygen concentration at position $\mathbf{x}$ is thus

$$\frac{\partial \omega}{\partial t} = D_\omega \nabla^2 \omega - \kappa \omega \sum_i \delta(\mathbf{x} - \mathbf{x}_i) \tag{S5}$$

where $\mathbf{x}_i$ is the location of cell i, and values of the dimensionless parameters κ and D_ω coincide with their dimensional equivalents due to our choice of timescales and lengthscales in Equation (S4). We note that for our choices of lengthscale and timescale the non-dimensional Equation (S5) and the dimensional Equation (S1) are identical, up to a rescaling of the oxygen concentration. We choose $\omega_0 = \omega_\infty$, to normalise concentration such that the oxygen concentration on the spheroid boundary in the non-dimensional system is 1.
