# Supplementary material for: Mathematical modelling reveals cellular dynamics within tumour spheroids
Source: PLoS Comput Biol. 2020 Aug 18;16(8):e1007961. doi: 10.1371/journal.pcbi.1007961 (PMC7455028; doi:10.1371/journal.pcbi.1007961)
Supplement: S4 Appendix — (PDF) [file pcbi.1007961.s004.pdf]

Supporting information

For: **Mathematical modelling reveals cellular dynamics within tumour spheroids**

by Joshua A. Bull, Franziska Mech, Tom Quaiser, Sarah L. Waters, Helen M. Byrne

S4 Appendix: Microbead infiltration histograms for other parameter combinations Fig B reproduces microbead infiltration histograms like those shown in Fig 2 and [1] for a range of representative parameter sets (averaged over 40 simulation repetitions each). For each parameter combination, the distribution takes the form of a initial peak at the spheroid boundary which gradually moves radially towards the spheroid centre, with the distribution dispersing over time. There is no clear way to determine which parameter combinations were used to generate each distribution by studying just these histograms, implying that spheroid composition cannot be easily inferred from microbead infiltration experiments in which individual microbeads cannot be resolved.

References

1. Dorie MJ, Kallman RF, Rapacchietta DF, Van Antwerp D, Huang YR. Migration and internalization of cells and polystyrene microspheres in tumor cell spheroids. Experimental Cell Research. 1982;141(1):201–209. doi:10.1016/0014-4827(82)90082-9.

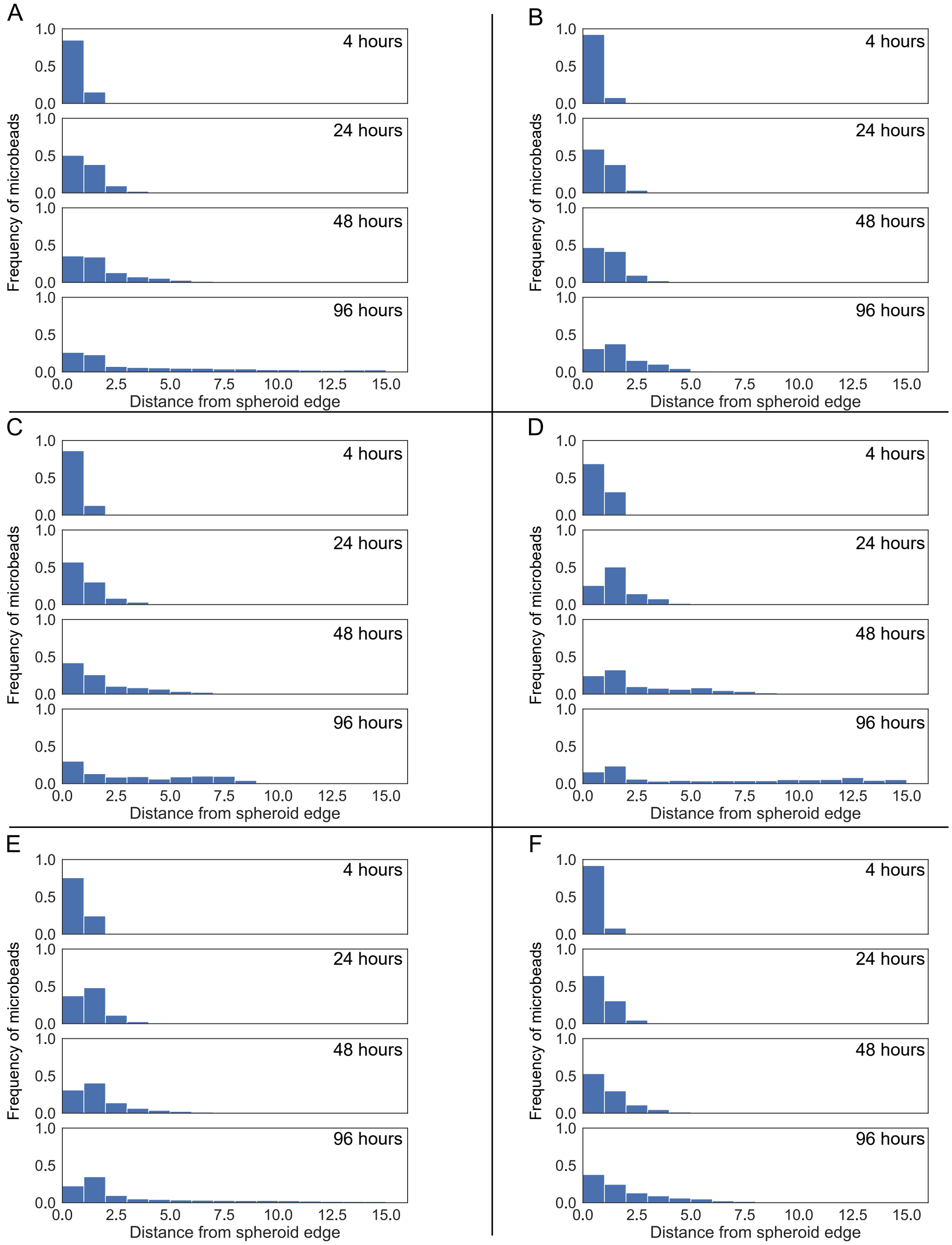

Fig B. Frequency histograms for microbead infiltration from a range of parameter sets.

Microbead infiltration histograms generated from tumour spheroid simulations with a range of parameter combinations, averaged over 40 simulation repetitions each. All combinations of parameters show an initial peak at the spheroid boundary, followed by eventual dispersion of the distribution towards the spheroid centre. Parameter combinations:

A: $\omega_{\mathrm{q}} = 0.5$, $\omega_{\mathrm{h}} = 0.3$, $\tau = 24$, $\tilde{\tau} = 8$;
B: $\omega_{\mathrm{q}} = 0.7$, $\omega_{\mathrm{h}} = 0.1$, $\tau = 32$, $\tilde{\tau} = 8$;
C: $\omega_{\mathrm{q}} = 0.7$, $\omega_{\mathrm{h}} = 0.7$, $\tau = 18.67$, $\tilde{\tau} = 16$;
D: $\omega_{\mathrm{q}} = 0.5$, $\omega_{\mathrm{h}} = 0.1$, $\tau = 13.33$, $\tilde{\tau} = 16$;
E: $\omega_{\mathrm{q}} = 0.3$, $\omega_{\mathrm{h}} = 0.1$, $\tau = 24$, $\tilde{\tau} = 8$;
F: $\omega_{\mathrm{q}} = 0.7$, $\omega_{\mathrm{h}} = 0.5$, $\tau = 32$, $\tilde{\tau} = 16$.
