# Supplementary material for: Mathematical modelling reveals cellular dynamics within tumour spheroids
Source: PLoS Comput Biol. 2020 Aug 18;16(8):e1007961. doi: 10.1371/journal.pcbi.1007961 (PMC7455028; doi:10.1371/journal.pcbi.1007961)
Supplement: S5 Appendix — (PDF) [file pcbi.1007961.s005.pdf]

Supporting information

For: **Mathematical modelling reveals cellular dynamics within tumour spheroids**
by Joshua A. Bull, Franziska Mech, Tom Quaiser, Sarah L. Waters, Helen M. Byrne

S5 Appendix: Replication of ^{3}H-labelled cells experiments Fig C shows the results of representative simulations for the infiltration of ^{3}H-labelled cells into tumour spheroids. Panel A shows data from [1] which describe the frequency of labelled cells as distance from the spheroid edge varies at 4 timepoints. The distribution of labelled cells moves radially inwards, but in contrast to the microbead data reproduced in Fig 3 the peak of the distribution remains at the spheroid edge. Panels B - F of Fig C show our simulations of ^{3}H-labelled cells from 5 representative parameter sets (30 repetitions each). Some parameter combinations closely match the distributions shown in [1], such as those shown in Panels B and C, while all distributions retain a similar pattern in which the distribution moves radially inwards while retaining its maximum close to the spheroid boundary.

An explanation for the difference between microbead infiltration and ^{3}H-labelled cell infiltration patterns is given by Fig D. Panel A reproduces Panel A of Fig C, while Panels B - F show the data in Panels B - F of Fig C without normalising the distributions. Through expressing the data as the total number of ^{3}H-labelled cells at a given radius, we can colour each bar according to the number of cells in each oxygen compartment. This demonstrates that, for a given parameter set, the population of proliferative ^{3}H-labelled cells gradually increases until it reaches a constant level. After this time, the number of proliferative cells remains constant while additional ^{3}H-labelled cells are pushed into the quiescent and hypoxic compartments. The apparent diffusion radially inwards of the distributions in Fig C is due to the effect of normalisation - as the population of ^{3}H-labelled cells increases, the size of the peak of proliferative cells at the spheroid edge appears to drop and the wave appears to move inwards while retaining a strong presence at the spheroid boundary. We conclude that the differences between distributions of microbeads and ^{3}H-labelled cells observed in [1] is due predominantly to the proliferation of ^{3}H-labelled cells at the spheroid edge. While proliferation of ^{3}H-labelled cells has be considered in previous models [2, 3], our model can successfully explain the behaviour of both microbead and ^{3}H-labelled cell distributions without requiring labelled cells to be initialised within the spheroid [2] or the postulation of additional chemotactic gradients driving the movement of tumour cells [3, 4].

Fig E demonstrates this process for two representative simulations generated using the parameter combinations of panels D and F in Fig C and Fig D. ^{3}H-labelled cells at the spheroid edge establish small colonies of labelled cells, whose offspring are carried along by advection in the cellular flow. This leads to a distinctive pattern of "spokes" at later timepoints in Fig E, where colonies of ^{3}H-labelled cells spread from the proliferative rim through to the necrotic core.

References

1. Dorie MJ, Kallman RF, Rapacchietta DF, Van Antwerp D, Huang YR. Migration and internalization of cells and polystyrene microspheres in tumor cell

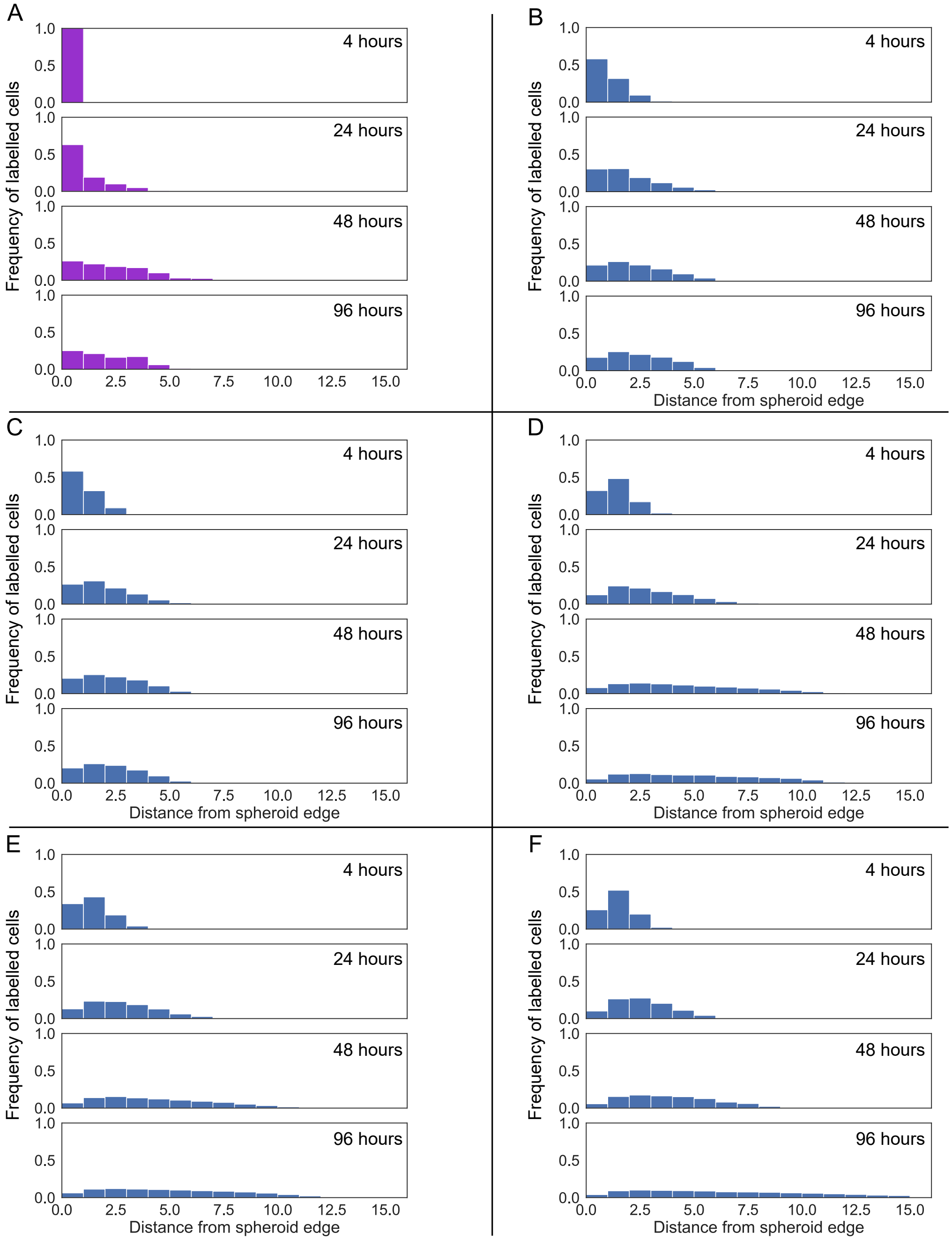

Fig C. Frequency histograms for ^{3}H-labelled cell infiltration from a range of parameter sets.

Frequency histograms for ^{3}H-labelled cell infiltration generated from tumour spheroid simulations with a range of parameter combinations, averaged over 30 simulation repetitions each. A: Data from Fig 6 in [1] showing infiltration of ^{3}H-labelled RIF-1 cells into tumour spheroids. B-F: simulated ^{3}H-labelled cell infiltration patterns for parameter sets:

B: $\omega_\text{q} = 0.7$, $\omega_\text{h} = 0.7$, $\tau = 32$, $\tilde{\tau} = 16$;

C: $\omega_\text{q} = 0.7$, $\omega_\text{h} = 0.7$, $\tau = 24$, $\tilde{\tau} = 8$;

D: $\omega_\text{q} = 0.5$, $\omega_\text{h} = 0.3$, $\tau = 16$, $\tilde{\tau} = 8$;

E: $\omega_\text{q} = 0.7$, $\omega_\text{h} = 0.3$, $\tau = 8$, $\tilde{\tau} = 8$;

F: $\omega_\text{q} = 0.7$, $\omega_\text{h} = 0.1$, $\tau = 8$, $\tilde{\tau} = 16$.

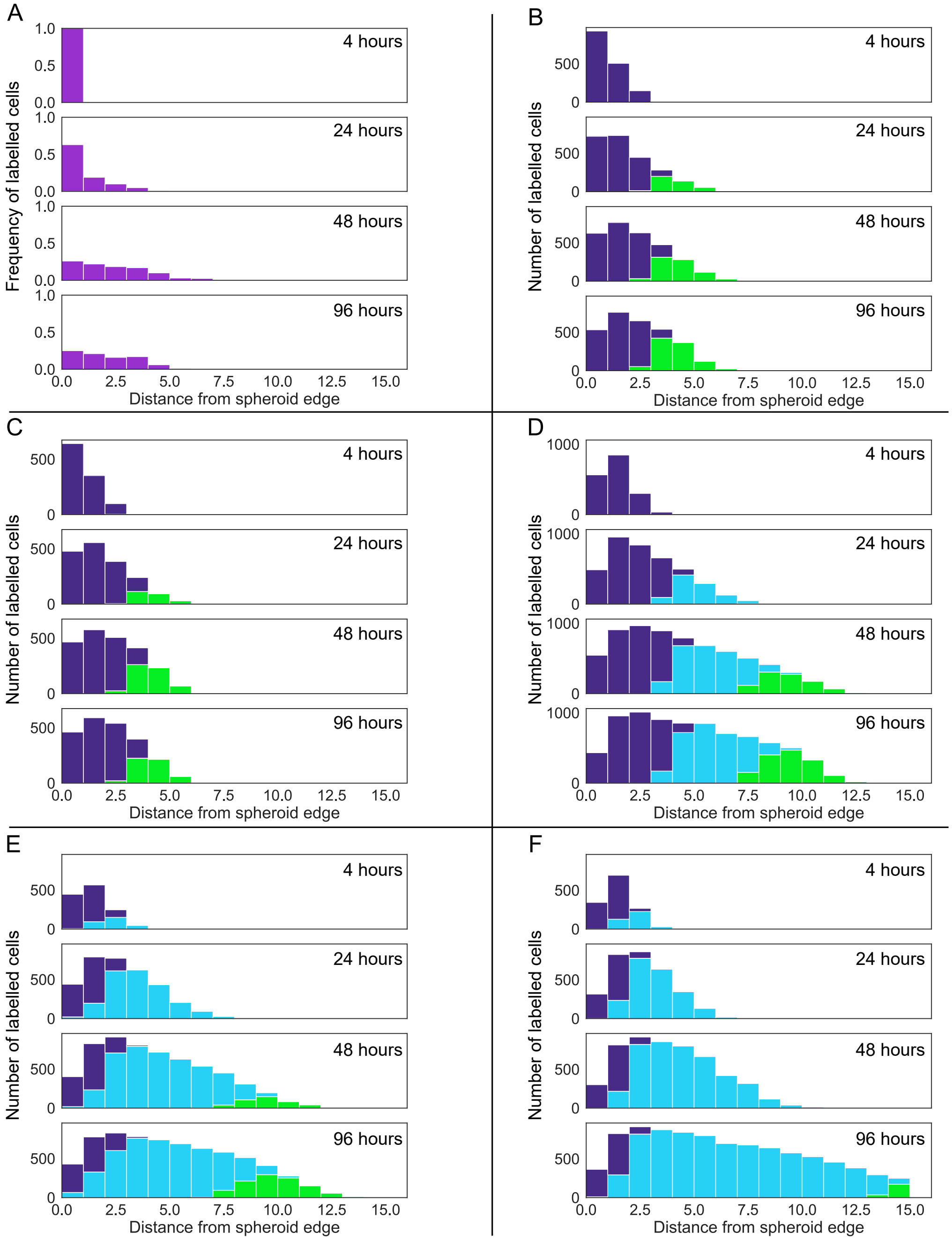

Fig D. Histograms showing total numbers of ^{3}H-labelled cells for the data shown in Fig C.
Data from the ^{3}H-labelled cell infiltration histograms shown in Fig C, showing the total number of ^{3}H-labelled cells without normalisation. Each bar is coloured according to the number of labelled cells of each oxygen phenotype at the relevant radial distance from the spheroid edge (necrotic cells lose their label, and so are not shown - colours as in Fig 1). Plotting the data in this way reveals that the number of ^{3}H-labelled cells in the proliferative rim eventually reaches saturation, and that the apparent radial infiltration observed in Fig C is due to an increasing number of ^{3}H-labelled cells in the quiescent and hypoxic regions rather than a decrease in the number of labelled cells at the spheroid edge. A: reproduction of Fig 6 from [1], B-F: Parameter values stated in caption for Fig C.

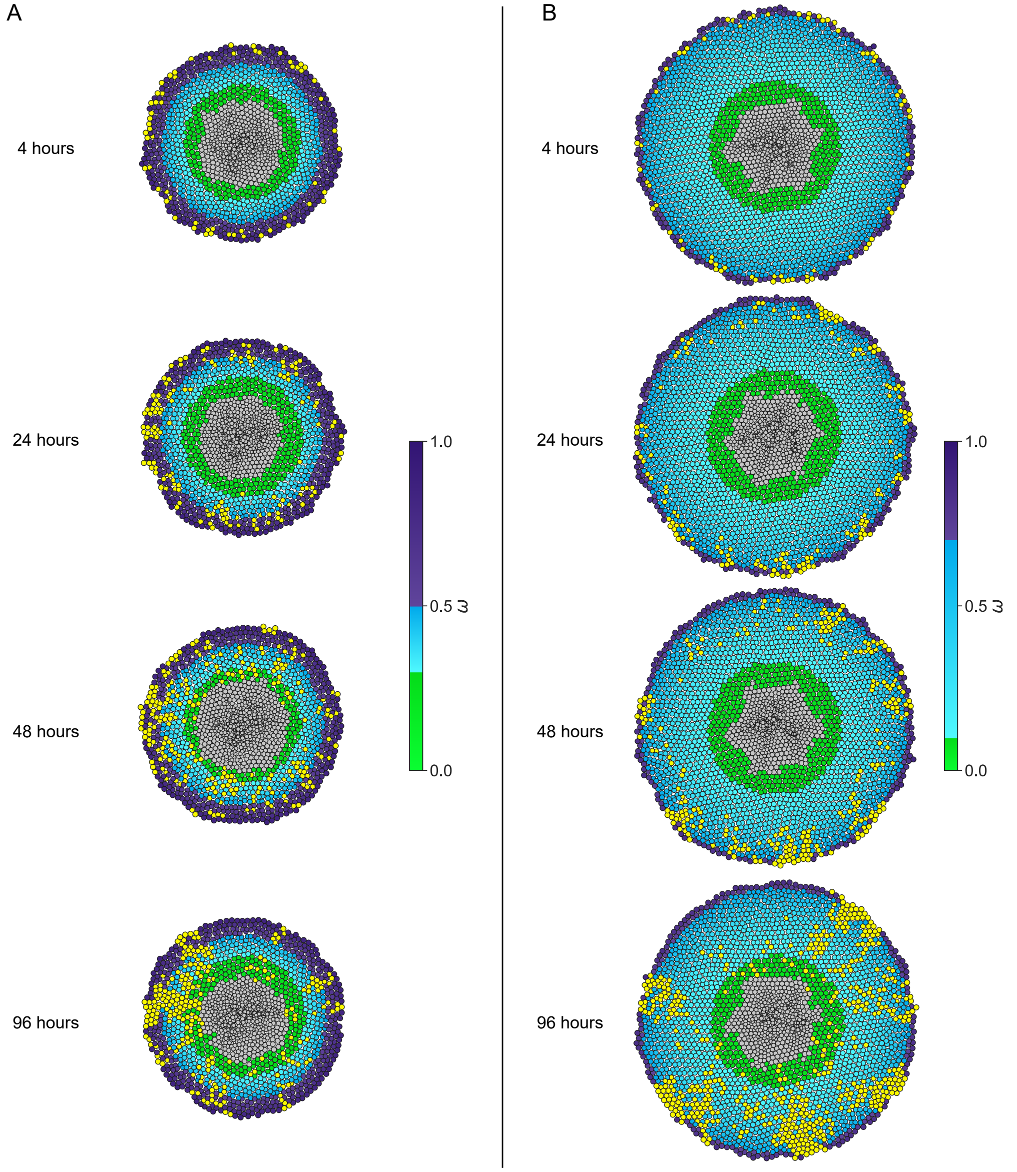

Fig E. Representative simulations of ^{3}H-labelled cell infiltration into tumour spheroids.
Snapshots from simulations of ^{3}H-labelled cells for two representative parameter sets. ^{3}H-labelled cells are coloured yellow regardless of their phenotype, although they behave according to the same rules as unlabelled cells. Proliferative ^{3}H-labelled cells close to the spheroid rim produce offspring which are carried radially inwards via advection by the cellular flow, causing infiltration of ^{3}H-labelled cells in clusters while the number of proliferative labelled cells remains approximately constant. ^{3}H-labelled cells lose their label when they become necrotic. Parameter sets: A: $\omega_{\mathrm{q}} = 0.5$, $\omega_{\mathrm{h}} = 0.3$, $\tau = 16$, $\tilde{\tau} = 8$ (panel D in Fig Fig C and Fig Fig D). B: $\omega_{\mathrm{q}} = 0.7$, $\omega_{\mathrm{h}} = 0.1$, $\tau = 8$, $\tilde{\tau} = 16$ (panel F in Fig Fig C and Fig Fig D)

spheroids. Experimental Cell Research. 1982;141(1):201–209. doi:10.1016/0014-4827(82)90082-9.

2. Thompson KE, Byrne HM. Modelling the internalization of labelled cells in tumour spheroids. Bulletin of Mathematical Biology. 1999;61(4):601–623. doi:10.1006/bulm.1999.0089.

3. Pettet GJ, Please CP, Tindall MJ, Mcelwain DLS. The migration of cells in multicell tumor spheroids. Bulletin of Mathematical Biology. 2001;63(2):231–257. doi:10.1006/bulm.2000.0217.

4. McElwain DLS, Pettet GJ. Cell migration in multicell spheroids: Swimming against the tide. Bulletin of Mathematical Biology. 1993;55(3):655–674. doi:10.1007/BF02460655.
