# Supplementary material for: Mathematical modelling reveals cellular dynamics within tumour spheroids
Source: PLoS Comput Biol. 2020 Aug 18;16(8):e1007961. doi: 10.1371/journal.pcbi.1007961 (PMC7455028; doi:10.1371/journal.pcbi.1007961)
Supplement: S6 Appendix — (PDF) [file pcbi.1007961.s006.pdf]

Supporting information

For: **Mathematical modelling reveals cellular dynamics within tumour spheroids**
by Joshua A. Bull, Franziska Mech, Tom Quaiser, Sarah L. Waters, Helen M. Byrne

S6 Appendix: Predicting spheroid composition from microbead observations In Fig 6 we demonstrated that nearest-neighbours classification allows the quiescent proportion of the tumour spheroid radius to be estimated accurately. Fig F shows that the proliferative, hypoxic and necrotic proportions of the spheroid radius can be inferred by observing T_{wait} and V_r in the same way.

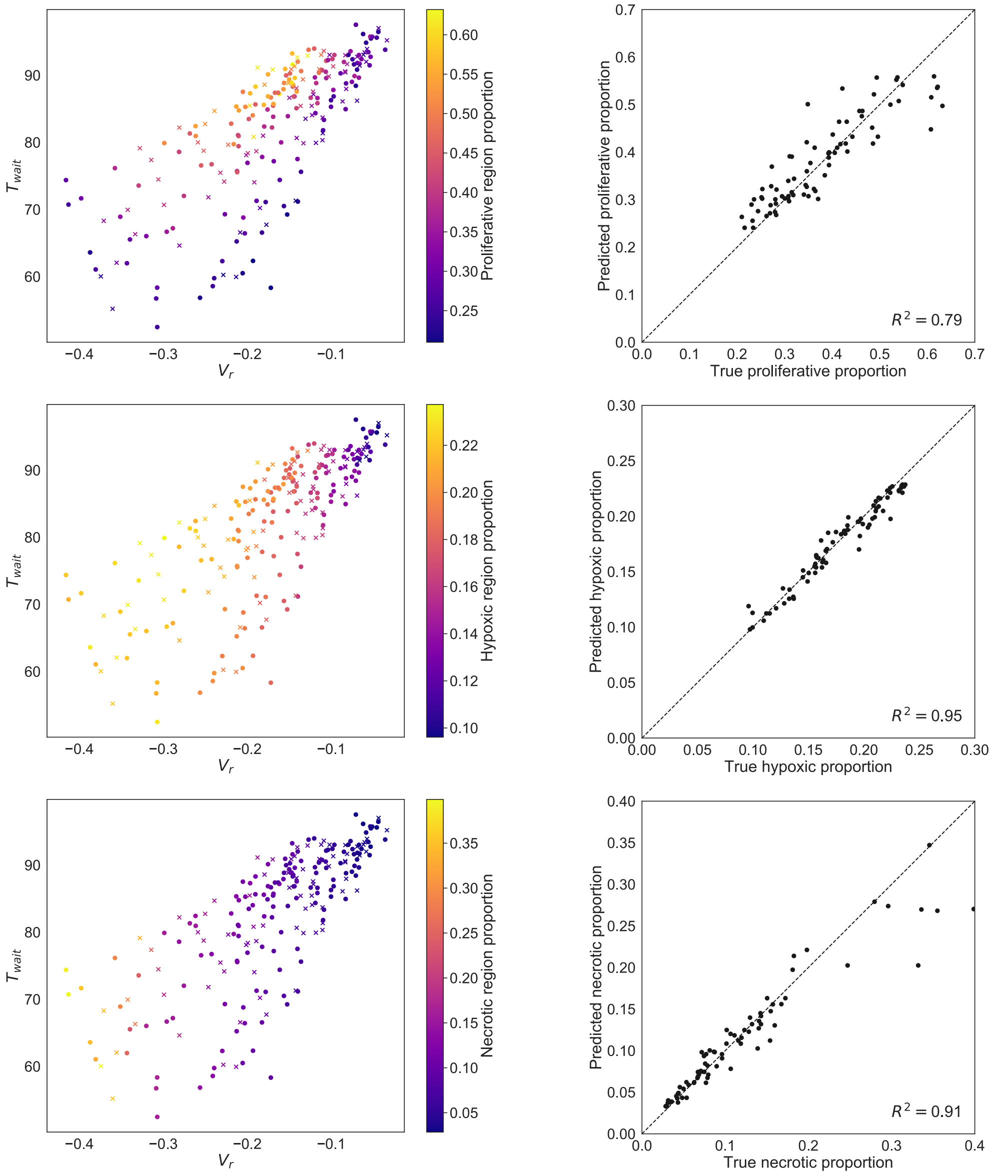

Fig F. Extension of Fig 6 to predict the proportion of the spheroid radius occupied by the proliferative, hypoxic and necrotic compartments.
