# Supplementary material for: Mathematical modelling reveals cellular dynamics within tumour spheroids
Source: PLoS Comput Biol. 2020 Aug 18;16(8):e1007961. doi: 10.1371/journal.pcbi.1007961 (PMC7455028; doi:10.1371/journal.pcbi.1007961)
Supplement: S7 Appendix — (PDF) [file pcbi.1007961.s007.pdf]

Supporting information

For: **Mathematical modelling reveals cellular dynamics within tumour spheroids**
by Joshua A. Bull, Franziska Mech, Tom Quaiser, Sarah L. Waters, Helen M. Byrne

S7 Appendix: Table of parameters Table A contains the default parameter values used in this model (ranges indicate parameters which are altered in different simulations). Where no reference is given, the parameter value has been chosen in order to produce biologically reasonable behaviour. In particular, parameter values were chosen to ensure that spheroids remained compact, exhibited logistic growth patterns characteristic of diffusion-limited spheroid growth and, where possible, were consistent with previous modelling studies. Dimensional values for parameters relating to oxygen thresholds are stated in terms of partial pressures p; following [1] and [2] these can be converted to concentrations ω using Henry's Law $p = \Omega\omega$, with $\Omega = 3.0318 \times 10^7$mmHg kg m^{-3}.

Table A. Parameter values and ranges used for simulations.

Symbol	Parameter	Dimensionless value	Dimensional range	Refs
dt	Timestep	1/120	1/200 - 1/100 (hours)	[3]
R_{Cell}	Radius of a cell	0.5	7 - 12 (μm)	[4]
R_{int}	Radius of interaction	1.5	21 - 36 (μm)	[3]
α	Radius used to determine α-shape	0.5	7 - 12 (μm)	*
ω_∞	Oxygen boundary value	1.0	100 - 150 (mm Hg)	[1, 5]
ω_{h}	Hypoxia threshold	0.1 - 0.7	10 (mm Hg)	[1]
ω_{q}	Quiescence threshold	0.3 - 0.7	30 - 70 (mm Hg)	*
τ	Average cell cycle length	8 - 32	13 - 32 (hours)	[6, 7]
τ_i	Cell cycle duration for cell i	0.75τ- 1.25τ	9.75 - 40 (hours)	*
$\tilde{\tau}$	Average critical hypoxic duration	8 - 16	Assumed (hours)	*
$\tilde{\tau}_i$	Critical hypoxic duration for cell i	$0.75\tilde{\tau}$- $1.25\tilde{\tau}$	Assumed (hours)	*
$\bar{\tau}$	Average necrosis duration	48	Assumed (hours)	*
$\bar{\tau}_i$	Necrosis duration for cell i	$0.75\bar{\tau}$- $1.25\bar{\tau}$	Assumed (hours)	*
ν	Damping coefficient	1	0.4 ($N\,\text{s}^{-1}\text{m}^{-1}$)	[3, 8]
μ	Spring constant	45.0	3 - 50 (μg Cell diameter^{-1} hours^{-2})	[3, 9]
μ_{bead}	Spring constant for microbeads	45.0	3 - 50 (μg Cell diameter^{-1} hours^{-2})	*
λ	Intercellular adhesion scaling coefficient	5.0	Assumed (-)	[3]
s_i	Radius of cell i at equilibrium	R_{Cell}	7 - 12 (μm)	[4]
$s_{i,j}$	Resting spring length between cells i and j	$s_i + s_j$	0 - 24 (μm)	*
D	Random motility coefficient	0.01	Assumed (Cell diameter^2 hours^{-1})	*
κ	Oxygen consumption rate	0.03	20×10^{-18} (mol/(cell s))	[10]
D_ω	Oxygen diffusion coefficient	1	1,750 (μm^2 seconds^{-1})	[10]
β	Surface tension coefficient	5	Assumed (μg hours^{-2})	*

*Estimated to maintain realistic model behaviour.

References

1. Grimes DR, Kelly C, Bloch K, Partridge M A method for estimating the oxygen consumption rate in multicellular tumour spheroids. Journal of the Royal Society Interface. 2014;11:20131124 doi:10.1098/rsif.2013.1124.
2. Lewin TD, Maini PK, Moros EG, Enderling H, Byrne HM. The Evolution of Tumour Composition During Fractionated Radiotherapy: Implications for Outcome. Bulletin of Mathematical Biology. 2018;80;1207–1235 doi:10.1007/s11538-018-0391-9.
3. Osborne JM, Fletcher AG, Pitt-Francis JM, Maini PK, Gavaghan DJ. Comparing individual-based approaches to modelling the self-organization of multicellular tissues. PLoS Computational Biology. 2017;13(2):1–34. doi:10.1371/journal.pcbi.1005387.
4. Laget S, Broncy L, Hormigos K, Dhingra DM, BenMohamed F, Capiod T, Osteras M, Farinelli L, Jackson S, Paterlini-Bréchot P. Technical Insights into Highly Sensitive Isolation and Molecular Characterization of Fixed and Live Circulating Tumour Cells for Early Detection of Tumor Invasion. PLoS ONE. 2017;12(1);e0169427 doi:10.1371/journal.pone.0169427.
5. Mueller-Klieser WF, Sutherland RM. Oxygen Tensions in Multicell Spheroids of Two Cell Lines. British Journal of Cancer. 1982;45(2);256–264 doi:10.1038/bjc.1982.41.
6. Jagiella N, Müller B, Müller M, Vignon-Clementel IE, Drasdo D. Inferring Growth Control Mechanisms in Growing Multi-cellular Spheroids of NSCLC Cells from Spatial-Temporal Image Data. PLoS Computational Biology. 2016;12(2) doi:10.1371/journal.pcbi.1004412.
7. Landry JL, Freyer JP, Sutherland RM. Shedding of Mitotic Cells From the Surface of Multicell Spheroids During Growth. Journal of Cellular Physiology. 1981;106;23–32 doi:10.1371/journal.pone.0080516.
8. Pathmanathan P, Cooper J, Fletcher A, Mirams G, Murray P, Osborne J, Pitt-Francis J, Walter A, Chapman SJ A computational study of discrete mechanical tissue models. Physical Biology. 2009;6(3) doi:10.1088/1478-3975/6/3/036001.
9. Dunn SJ, Näthke IS, Osborne JM. Computational Models Reveal a Passive Mechanism for Cell Migration in the Crypt. PLoS ONE. 2013;8(11);1–18 doi:10.1371/journal.pone.0080516.
10. Schaller G, Meyer-Hermann M Multicellular tumor spheroid in an off-lattice Voronoi-Delaunay cell model. Physical Review E. 2005;71(5);1–16 doi:10.1103/PhysRevE.71.051910.
