# Supplementary material for: Mathematical modelling reveals cellular dynamics within tumour spheroids
Source: PLoS Comput Biol. 2020 Aug 18;16(8):e1007961. doi: 10.1371/journal.pcbi.1007961 (PMC7455028; doi:10.1371/journal.pcbi.1007961)
Supplement: S8 Appendix — (PDF) [file pcbi.1007961.s008.pdf]

Supporting information

For: **Mathematical modelling reveals cellular dynamics within tumour spheroids**
by Joshua A. Bull, Franziska Mech, Tom Quaiser, Sarah L. Waters, Helen M. Byrne

S8 Appendix: Impact of initial spheroid radius on steady state dynamics
In Fig G we show how changing the initial spheroid radius impacts the subsequent growth dynamics, for the same parameter set used in Figure 2 ($\tau = 16$, $\omega_{\mathrm{h}} = 0.3$, $\omega_{\mathrm{q}} = 0.5$, $\tilde{\tau}_i = 8$). For each case, we show the mean and standard deviation of 10 realisations.

Each simulated spheroid attains the same steady state radius and spatial composition, indicating that our simulations are robust to changes in the initial conditions. We note that the duration of the burn-in period, before the growth curves resemble a sigmoid function, increases as the initial radius of the spheroid increases. During this period quiescent, hypoxic and necrotic regions, which are not present at $t = 0$, are established. Further, as a cell must remain hypoxic for $\tilde{\tau}_i$ hours before it can become necrotic, spheroids which are initially large enough to sustain a necrotic core will not exhibit necrosis until this time. We note that in all cases the steady state size has been reached by $t = 300$. Therefore in microbead simulations, microbeads are added to the spheroids at $t = 300$.

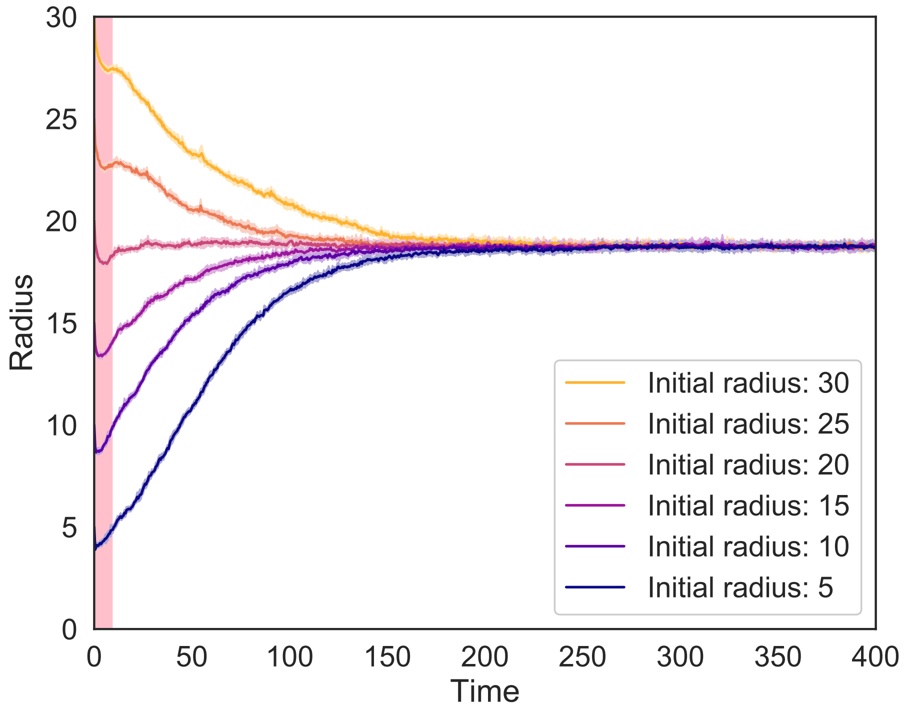

Fig G. Simulation of spheroid growth for the parameter set used in Fig 2: $\tau = 16$, $\omega_{\mathrm{h}} = 0.3$, $\omega_{\mathrm{q}} = 0.5$, $\tilde{\tau}_i = 8$. Simulations were initialised with cell clusters of between 5 and 30 cell diameters in radius. For each choice of initial condition, 10 realisations were generated (mean and SD shown by solid lines and shaded areas respectively). All spheroids attained the same steady state radius and spatial composition. The red shaded area represents a burn-in period, whose duration increases as the initial size of the spheroid increases, due to the time taken to establish hypoxic and necrotic zones.
