# Supplementary material for: Mathematical modelling reveals cellular dynamics within tumour spheroids
Source: PLoS Comput Biol. 2020 Aug 18;16(8):e1007961. doi: 10.1371/journal.pcbi.1007961 (PMC7455028; doi:10.1371/journal.pcbi.1007961)
Supplement: S9 Appendix — (PDF) [file pcbi.1007961.s009.pdf]

Supporting information

For: **Mathematical modelling reveals cellular dynamics within tumour spheroids**
by Joshua A. Bull, Franziska Mech, Tom Quaiser, Sarah L. Waters, Helen M. Byrne

S9 Appendix: schematic of forces acting on cells Fig H shows how the forces acting on the tumour cells varies with location within a simulated tumour spheroid (for parameters $\tau = 32$, $\omega_{\mathrm{h}} = 0.3$, $\omega_{\mathrm{q}} = 0.7$, $\tilde{\tau}_i = 16$). The mechanical forces arise from physical interactions between a cell and cells within its interaction radius. We consider (i) a cell on the spheroid boundary (top left inset), (ii) cells in the quiescent region (top right inset experiencing net outward force, bottom left inset experiencing net inward force), and (iii) a cell at the edge of the necrotic core (bottom right inset).

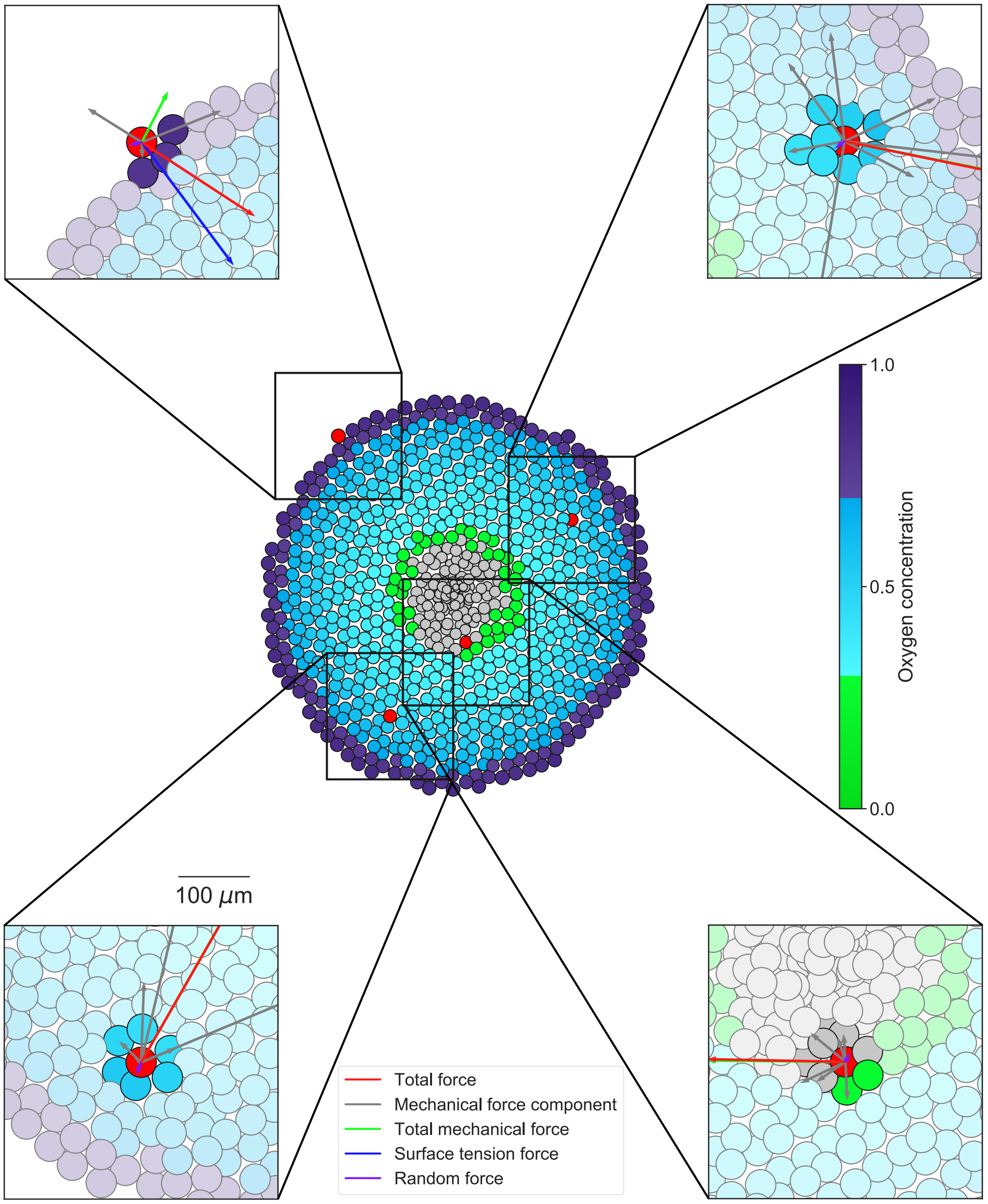

Fig H. Summary of individual forces acting on four tumour cells (coloured red) at different spatial locations within the same tumour spheroid which has attained its equilibrium size and composition (with parameter values $\tau = 32$, $\omega_{\mathrm{h}} = 0.3$, $\omega_{\mathrm{q}} = 0.7$, $\tilde{\tau}_i = 16$). In the inset images, cells which are outside the radius of interaction of the marked cell are shown as faded.
Top left inset: Cell on the spheroid boundary.
Top right inset: Cell in the quiescent region, experiencing net force away from the spheroid core.
Bottom left inset: Cell in the quiescent region, experiencing net force towards the spheroid core.
Bottom left inset: Cell at the edge of the necrotic region.
